# 3D stem-like spheroids-on-a-chip for personalized combinatorial drug testing in oral cancer

**DOI:** 10.1186/s12951-024-02625-y

**Published:** 2024-06-18

**Authors:** Viraj Mehta, Sukanya Vilikkathala Sudhakaran, Vijaykumar Nellore, Srinivas Madduri, Subha Narayan Rath

**Affiliations:** 1 Regenerative Medicine and Stem Cell Laboratory (RMS), Department of Biomedical Engineering, Indian Institute of Technology Hyderabad, Sangareddy, Kandi, 502285 Telangana India; 2 Department of Surgery, University of Geneva, 1205 Geneva, Switzerland

**Keywords:** Spheroids-on-chips, Personalized medicine, 3D printing, Combinatorial drug testing, Functional drug testing, Microfluidics

## Abstract

**Background:**

Functional drug testing (FDT) with patient-derived tumor cells in microfluidic devices is gaining popularity. However, the majority of previously reported microfluidic devices for FDT were limited by at least one of these factors: lengthy fabrication procedures, absence of tumor progenitor cells, lack of clinical correlation, and mono-drug therapy testing. Furthermore, personalized microfluidic models based on spheroids derived from oral cancer patients remain to be thoroughly validated. Overcoming the limitations, we develop 3D printed mold-based, dynamic, and personalized oral stem-like spheroids-on-a-chip, featuring unique serpentine loops and flat-bottom microwells arrangement.

**Results:**

This unique arrangement enables the screening of seven combinations of three drugs on chemoresistive cancer stem-like cells. Oral cancer patients-derived stem-like spheroids (CD 44^+^) remains highly viable (> 90%) for 5 days. Treatment with a well-known oral cancer chemotherapy regimen (paclitaxel, 5 fluorouracil, and cisplatin) at clinically relevant dosages results in heterogeneous drug responses in spheroids. These spheroids are derived from three oral cancer patients, each diagnosed with either well-differentiated or moderately-differentiated squamous cell carcinoma. Oral spheroids exhibit dissimilar morphology, size, and oral tumor-relevant oxygen levels (< 5% O_2_). These features correlate with the drug responses and clinical diagnosis from each patient’s histopathological report.

**Conclusions:**

Overall, we demonstrate the influence of tumor differentiation status on treatment responses, which has been rarely carried out in the previous reports. To the best of our knowledge, this is the first report demonstrating extensive work on development of microfluidic based oral cancer spheroid model for personalized combinatorial drug screening. Furthermore, the obtained clinical correlation of drug screening data represents a significant advancement over previously reported personalized spheroid-based microfluidic devices. Finally, the maintenance of patient-derived spheroids with high viability under oral cancer relevant oxygen levels of less than 5% O_2_ is a more realistic representation of solid tumor microenvironment in our developed device.

**Graphical Abstract:**

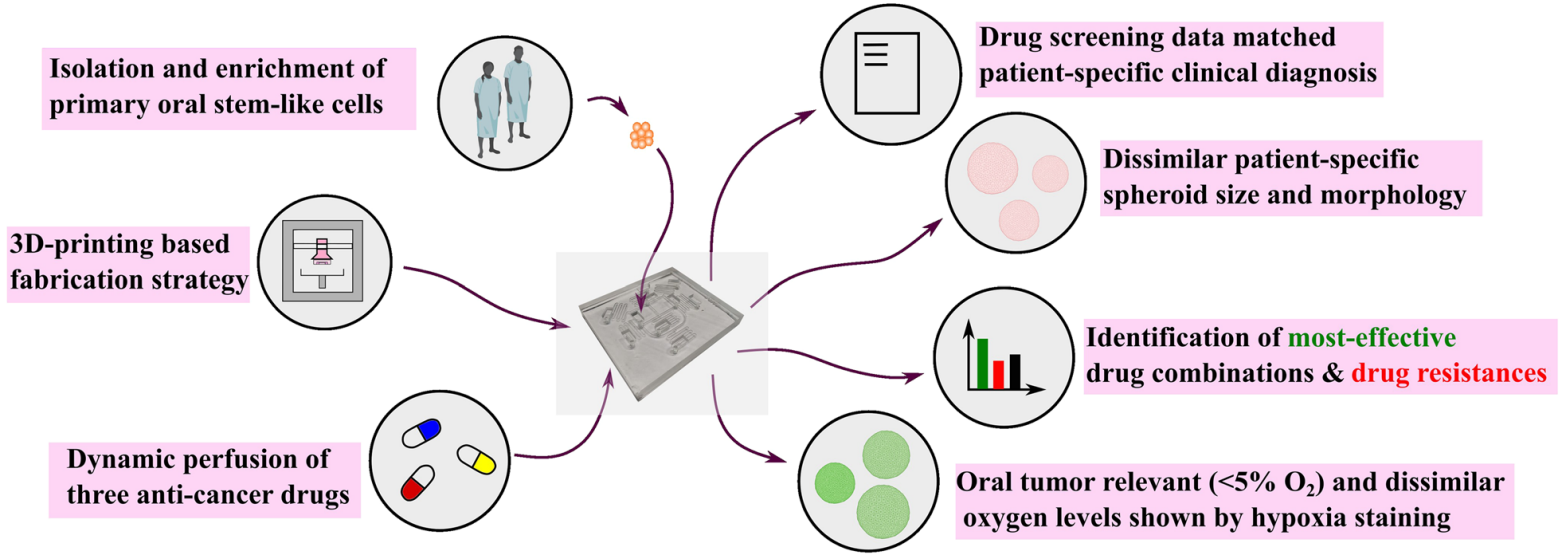

**Supplementary Information:**

The online version contains supplementary material available at 10.1186/s12951-024-02625-y.

## Background

The rising trend in cancer treatment failure and cancer deaths is partly due to the molecular and physical cancer heterogeneity and *one-size-fits-all* cancer treatment approach [1, 2]. The personalized and precision medicine approach aims to address the heterogeneous nature of cancer by providing the most suitable therapy for each individual patient [3]. The functional drug testing (FDT) approach in precision medicine is dissimilar to the DNA sequencing approach in which various drug molecules are tested on patient-derived tumor samples. Functional approaches can potentially guide the initial treatment decisions and predict the treatment outcomes [4].

Head & neck cancer is among the five most common cancers in India, accounting for approximately 40% of all cancers [5]. This asserts an urgent need to develop FDT models on patient-derived oral tumor samples. As cancer cells are known to evade drug mechanism pathways, combinations of drugs with dissimilar mechanisms of action can be beneficial over mono-drug therapies [6]. Functional approaches in oral cancer can aid in finding potential drug combinations.

One of the widely used FDT models is based on 3D tumor aggregates or spheroids. Microfluidic-based 3D spheroid models enable drug testing with a minimal amount of patient samples, maintain dynamic delivery of drug molecules and retain endogenous cell signaling molecules [7–9]. However, a significant number of the previously reported microfluidic drug testing models based on 3D spheroids were limited by the use of non-patient-specific cells [10–13]. Hence, patient-derived tumor spheroids-based microfluidic models emerged for FDT. Previously reported primary tumor spheroids-based FDT models have been greatly useful [14, 15]. However, static drug treatments, mono-drug therapy testing, lack of clinical correlation and longer fabrication procedures are some of the limitations that need to be addressed. Besides, the model should include cancer stem-like progenitor cells, as they are responsible for chemoresistance and tumor relapse.

Personalized microfluidic models in head & neck cancer remain to be thoroughly validated, as there have been very few reports involving 3D culture of oral tumor cells in the past decade [16–18]. Although these models offered patient-specific drug testing results, they lacked conclusive clinical correlation of observed drug responses [16–18]. Furthermore, they didn’t evaluate the oxygen levels in in vitro tumor models, which is known to affect the FDT readout [19, 20]. Besides, microfluidic models based on patient-derived stem-like spheroids are absent in head & neck cancer. Addressing the above challenges, we present a personalized, 3D printed soft-lithography mold-based, and dynamic oral stem-like *spheroid* model. This model is capable of testing seven combinations of three anti-cancer drugs simultaneously. The device features a distinctive design, incorporating seven serpentine loops that facilitate the mixing of drug combinations prior to their introduction to the 3D spheroid areas. For the creation of these 3D spheroids, arrays of seven cylindrical microwells are strategically positioned at the end of each serpentine loop.

Photolithography has been widely used for fabricating microfluidic channels with excellent surface finish and dimensional accuracy. However, 3D-printed mold-based approach has emerged as a potential competitor due to its several benefits over photolithography [21, 22]. Therefore, we evaluated and compared various photocurable 3D printing resins for fabricating spheroids-on-a-chip with replica molding in this study.

We hypothesize that the designed microfluidic device will be capable of identifying the most effective and suitable drug combinations for treatment of each oral cancer patient. Here, we evaluate the feasibility of using tumor-on-chips for personalized medicine. First, we presented the design of the device with computational validation. A cell seeding strategy was then developed to ensure uniform-sized spheroids formation throughout the seven microwell arrays. Next, we compared the optical transparency, surface roughness, and dimensional accuracy of polydimethylsiloxane (PDMS) devices produced from various 3D printed soft-lithography molds. Following the comparison of various photocurable resins, the device fabrication strategy was finalized. Next, we performed mixing characterization and surface characterization of the device. Subsequently, the devised cell seeding strategy and fabrication method were tested with MCF 7 cell line. Finally, we isolated oral tumor stem-like cells from biopsy samples of three oral cancer patients and characterized them with well-known cancer stem cell markers. Next, we demonstrated FDT with a well-known oral cancer chemotherapy regimen, including paclitaxel, 5 fluorouracil, and cisplatin on oral spheroids. More importantly, we selected maximum plasma concentrations of drugs (*C*_*max*_) associated with clinically recommended dosages from phase I/II clinical trials. Using *C*_*max*_, we aimed to generate a similar effect of drugs on spheroids, as experienced by native tumors at maximum plasma concentration. Here, we assume that drug concentration in tumor tissue at a pharmacokinetic steady state will be the same as drug concentration in plasma [23]. Finally, we presented clinical correlation of observed drug responses based on hypoxia dye staining, morphology, and size of patient-derived spheroids.

## Materials and methods

### Computational simulations

Velocity simulations were carried out in COMSOL Multiphysics laminar flow module. The density and dynamic viscosity of fluid was 993 kg/m^3^ and 8.9 × 10^–4^ Pa.s, respectively [24]. Boundary conditions were: inlet gauge pressure = 0 Pa, laminar outflow rate = 2 µl/min, and no-slip wall. Mixing simulation was carried out in the transport of diluted species module with convection and diffusion transport mechanisms. The isotropic diffusion coefficient of chemical species was considered 3 × 10^–10^ m^2^/s. This value represents the diffusion coefficient of paclitaxel, which has the highest molecular weight (853.906 g/mol) among the three drugs tested in this study. Steady-state simulations were carried out for velocity and mixing. Mixing simulation was carried out to determine the ideal length of a serpentine loop. In the simulation, we assigned 1.3% (v/v) concentration of species at one inlet and 0% (v/v) concentration at another inlet of the serpentine loop. We considered a linear relationship between concentration and mixing (%) to calculate mixing (%), as mentioned in Eq. 1. The mixing (%) and concentration of species were plotted at the end of the serpentine loop along a 300 µm vertical line.

Transient simulations were carried out for 800 s with a step size of 10 s for studying chemical species transport in microwells. At the inlet, 1% (v/v) arbitrary concentration of species was considered for the mass transport simulation. Spheroids of 100 µm size were shown at the bottom of the microwells.

1 $$\begin{gathered} m\, = \,\frac{c}{0.0065}\left( {0 \le c \le 0.65} \right) \hfill \\ m\, = \,\frac{{\left( {c\, - \,1.3} \right)}}{{\left( { - \,0.0065} \right)}}\left( {0.65 \le c \le 1.3} \right) \hfill \\ \end{gathered}$$

where, *m* = mixing(%), *c* = concentration of chemical species.

### Comparison of resins and fabrication of devices

Table 1 lists the 3D printing apparatus, type of technology, horizontal resolution, and layer height for each resin compared in the present study. Optical transmittance, dimensional accuracy, and surface roughness of PDMS devices were compared to select suitable photocurable resin material for fabricating soft-lithography mold of the microfluidic device. After selecting the resin material, PDMS spheroids-on-chips were fabricated using replica molding method (Figure S1). More details are provided in supplementary material and methods.

**Table 1:** List of resins for comparative study

3D printing apparatus	Type of technology	XY resolution	Resin	Layer height
3D Systems ProJet 6000 HD	Stereolithography (SLA)	4000 DPI (6.35 µm)	Accura 25, Accura ClearVue	Accura 25 (100 µm), Accura ClearVue (50 µm)
3D Systems FabPro 1000	Digital light processing (DLP)	65 µm	Proto GRY	50 µm
3D Systems ProJet MJP 2500	MultiJet printing (MJP)	1600 × 900 DPI (15.8 × 28.2 µm)	VisiJet M2R- CL	32 µm
Formlabs Form 3B	Low force stereolithography (LFS)	25 µm	Standard clear	25 µm

### Surface characterization

PDMS piece (0.5 × 1 cm) fabricated from a standard clear resin device was used. XPS analysis (AXIS Supra, Shimadzu, Kyoto, Japan) was carried out on a physically coated sample (2% (w/v) pluronic F127, 24 h) and a non-coated PDMS sample with an Al Kα X-ray source. X-ray power was 225 W. C1s spectra were corrected by adjusting C–C peak to 284.8 eV (+ 4.2 eV) [25]. A survey scan was obtained from 0 to 1200 eV with a 1 eV step size. Peaks were fitted using the Guass-Lorentz algorithm.

PDMS piece (0.8 × 1 cm) fabricated from a standard clear resin device was used. Contact angle goniometer (Rame-Hart Instrument Company, Succasunna, NJ, Model 290-F4) was used to measure the contact angle of 3 µl deionized water droplet on physically coated (2% (w/v) pluronic F127, 24 h) and native PDMS sample at 30 s intervals for 7 min.

MCF-7 cells (NCCS, Pune, India) were cultured in T75 flask in RPMI 1640 media (PAN-Biotech, Germany) supplemented with 10% FBS (fetal bovine serum, Gibco, Brazil) and 1% penicillin–streptomycin (Invitrogen, Thermo Fisher Scientific) at standard incubation conditions. The cells were trypsinized (0.25% Trypsin–EDTA, Sigma-Aldrich) at 70–80% confluency and seeded in a microfluidic device to evaluate anti-adhesion property of physically coated (2% (w/v) pluronic F127, 24 h) and native PDMS.

### Primary oral tumor stem-like cell isolation & characterization

The current study involving human samples was approved by the Institutional Ethics Committee (IEC) of IIT Hyderabad (IEC protocol number: IITH/IEC/2022/04/03). Oral tumor samples from untreated three patients (Table 2) were transported in appropriate medium after obtaining patient’s consent in a written format. Isolation of the primary oral tumor cells was performed according to the previously optimized enzymatic digestion procedure, as shown in Figure S2 [26]. Isolated oral cancer cells were grown in orosphere culture format for enrichment of stem-like cells. Finally, the stem-like properties of enriched cultures were evaluated with flow cytometry (CD44^High^/CD24^Low^) and immunofluorescence (ALDH1, EpCAM, and αSMA). Additional details of staining procedure can be found in supplementary material.

**Table 2:** Oral cancer patient details

Patient age (years)	Sex	Tumor staging (TNM)	Diagnosis
Patient 1 (63)	Female	T3N1M0	Well-differentiated squamous cell carcinoma
Patient 2 (46)	Male	T4N1M0	Well-differentiated squamous cell carcinoma
Patient 3 (60)	Female	T3N1M0	Moderately differentiated squamous cell carcinoma

### Cell culture in the device

The fabricated device was coated with 2% (w/v) pluronic F127 at room temperature for 24 h. The next day, the device was sterilized with UV and 70% ethanol solution for 10 min. Ethanol solution was replaced with cell culture medium before introducing 6000 cells/µl (20 µl cell suspension) from all seven inlets at a flow rate of 12 µl/min using 1 ml tips (minimum number of cells required to run device = 1 M/ml). Based on the input cell concentration of 6000 cells/µl/microwell array, the estimated number of cells captured in each microwell with a volume of 0.05 µl will be approximately 300 cells. Next, the microfluidic device was cultured at standard incubation conditions for 24 h for spheroid formation. Cell culture medium (DMEM + 20% FBS + growth factors) was exchanged two times in a day using gravity-based fluid flow for primary oral tumor cells. Besides, RPMI 1640 medium + 10% FBS was used to culture MCF-7 cells and exchanged two times a day. In the end, a cell viability assay was performed for MCF-7 spheroids and primary oral spheroids. We measured the size and circularity of spheroids from brightfield images using the “trainable weka segmentation” plugin in ImageJ. We took high-magnification images of spheroids and first cropped the area around the spheroids to eliminate the unnecessary background. Next, we trained the model by defining the leftover background signal and spheroids in the brightfield image. Finally, we obtained the spheroids separated from the background using the trained model. We next measured the segmented area and converted it to the diameter. The circularity was measured by $4\pi \left( {\frac{Area}{{perimeter^{2} }}} \right)$.

### Cell viability assay

Spheroids were stained with 1 µg/ml Calcein/AM (Invitrogen) and 4 µg/ml PI (propidium iodide, Molecular Probes, USA) solution prepared in 1 ml PBS. Quantification was performed using a previous protocol based on area fraction [27]. Individual spheroid images were cropped with 300 × 300 µm window size, and the area fractions of green and red channels were measured individually. Viability (%) was calculated according to the Eq. 2 using ImageJ.

2 $$Viability\,\left( \% \right)\, = \,\frac{{Area\,f\,raction_{green} }}{{Area\,f\,raction_{green} \, + \,Area\,f\,raction_{red} }}$$

### Immunofluorescence in device

On day 3, spheroids were fixed with 4% (v/v) formaldehyde solution for 30 min at room temperature. Next, spheroids were washed with 1 × PBS and permeabilized with 0.2% Triton X-100 (Sigma-Aldrich) solution for 1 h at room temperature. This was followed by incubation with 3% (w/v) BSA solution in 1 × PBS at 4 °C for 12–16 h to prevent non-specific binding. Next, spheroids were incubated with FITC conjugated anti-human CD 44 antibody (Elabscience TX, USA) (1:200) for 1 h at room temperature. Subsequently, spheroids were washed with 1 × PBS and counterstained with DAPI (1:1000) for 10 min before capturing the fluorescent images.

### Staining with hypoxia reagent

Image iT-green hypoxia reagent (Thermo-Fisher) at 1 µM was prepared in a cell culture medium and added to the device on day 3 for each patient-derived spheroids. Image iT-green hypoxia reagent detects oxygen levels less than 5% in cells, and its intensity is inversely proportional to the oxygen levels. The spheroids were incubated for 1 h before taking images in the microscope. All the images were taken at equal brightness settings in the microscope, and intensity was reported by subtracting the mean background fluorescence intensity measured from ImageJ.

### Drug perfusion

We selected maximum plasma concentrations of drugs (*C*_*max*_) associated with clinically recommended dosages from phase I/II clinical trials. Table 3 lists the referred phase I/II clinical trials for selecting the same. Well-known oral cancer regimen including paclitaxel (PTX) (T1912, Sigma-Aldrich) (1 µM) [28, 29], 5 fluorouracil (5 Fu) (F6627, Sigma-Aldrich) (20 µM) [30, 31], and cisplatin (CDDP) (P4394, Sigma-Aldrich) (5 µM) [32, 33] was dynamically withdrawn from three inlet ports towards four outlet ports at a flow rate of 2 µl/min on day 3. Paclitaxel suffers from low solubility in physiological buffers. Hence, DMSO based stock solutions were prepared according to our previous protocol and manufacturer’s instructions [34]. Working concentrations were prepared in cell culture medium with 1:1000 dilution, maintaining the final DMSO concentration < 0.1%. Firstly, silicon tubes were attached at four outlets, and 1 ml tips (filled with three drugs) were attached to the three inlets. Next, the syringe pump assembly connected with the device was kept in an incubator for 6 h under standard incubation conditions. The syringe pump applied negative pressure to withdraw the drugs from three inlets towards four outlets at 2 µl/min. Next, drugs were washed out by introducing cell culture medium, and the device was kept in static incubation for 2 h before performing the cell viability assay. Control cells were treated with 0.1% DMSO. The combination index (*CI*) was estimated by Eq. 3 [35, 36].

3 $$CI\, = \,\frac{{D_{1} }}{{D_{x1} }}\, + \,\frac{{D_{2} }}{{D_{x2} }}$$

where, *D*_*1*_ and *D*_*2*_ are drug concentrations of two drugs producing *z*% viability in combination and *D*_*x1*_ and *D*_*x2*_ are concentrations of two drugs in monotherapy producing same *z*% cell viability. The *CI* = 1 indicates the additive effect, *CI* < 1 indicates the synergistic effect and *CI* > 1 indicates the antagonistic effect of drugs in combination [35].

**Table 3:** Maximum plasma concentration selected for each drug

Drug	Maximum tolerated dosage (mg/m^2^)	Maximum plasma concentration (µM) and (infusion time)	Selected concentration (µM)	References
Paclitaxel	250	0.77–0.97 (3 or 24 h)	1	[28, 29]
5 fluorouracil	2600	20 (24 h), 15–23 (8 h)	20	[30, 31]
Cisplatin	80–100	6.3 ± 0.8 (24 h), 7.37 ± 3 (2 h), 4.62 ± 2.62 (4 h)	5	[32, 33]

### Statistical analysis

Graphpad Prism (GraphPad Software Inc., San Diego, CA, USA) software was used to perform a one-way analysis of variance (ANOVA) followed by Tukey’s post hoc to check the significance level. Significance was determined by *p* < 0.05 (*), *p* < 0.01(**), *p* < 0.001 (***). For each resin, two soft-lithography molds (*n* = 2) were used for channels and microwells measurement. The results were presented as Mean ± SD (standard deviation). In surface characterization, three PDMS devices (*n* = 3) were used for contact angle measurement, and the results were presented as Mean ± SD. The mixing experiment was repeated four times (*n* = 4), and mean values were obtained. They were presented as Mean ± SEM. The mean gray values for green dye solutions were compared using Student’s t-test. Spheroid size, circularity, and viability were reported as Mean ± SD with *n* = 49 spheroids from a device. Drug screening results were normalized to the control and presented as Mean ± SD with *n* = 7 spheroids from a device.

## Results

Figure S3 shows the overall sequence in which experiments for the tumor-on-chip were performed and presented in the manuscript. Since this is a proof-of-concept study, it involves significant biological data and engineering aspects, including design, fabrication and simulation. We have arranged the data in four major sections as shown in Figure S3: (1) Design, simulation & fabrication, (2) Device characterization, (3) Biological validation, and (4) Correlation with clinical data. Sections "Microfluidic device design and computational validation" & "Comparison of photocurable resins" present data from device design, fabrication and simulation. Section " Device characterization and validation with tumor cell line" presents data from device characterization. Section "Primary oral tumor stem-like cells isolation, characterization, and culture in the device" & "Personalized combinatorial drug screening" present data from biological validation with culture of patient-derived oral tumor stem-like spheroids and personalized drug screening. Section 3.6 & 3.7 present data of morphological evaluation and oxygen level evaluation in patient-derived tumor spheroids to correlate the drug testing data with clinical histopathological data.

### Microfluidic device design and computational validation

**Fig. 1:**
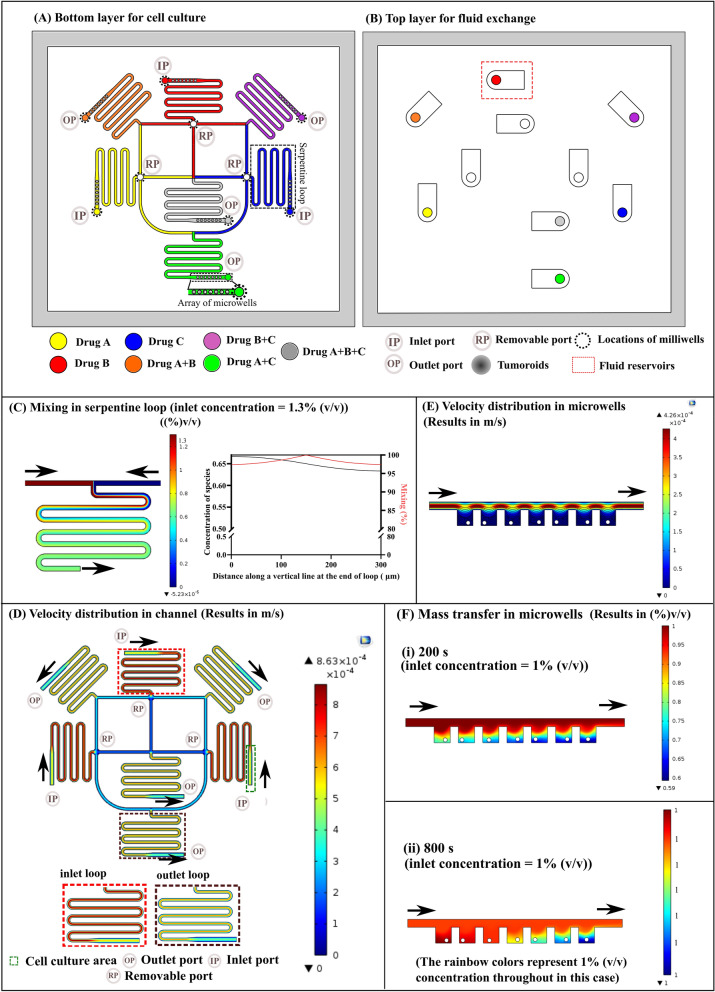
**A** Schematic of the bottom layer for cell culture with drug perfusion scheme. **B** Schematic of top layer for fluid exchange. **C** Mixing simulation in the serpentine loop of 57 mm length showing complete mixing at the end of the loop along a 300 µm vertical line with inlet concentration of species: 1.3% (v/v). **D** Velocity profile in channel network showing the *V*_*max*_ in inlet and outlet cell culture area. **E** Velocity profile with velocity streamlines in microwells. **F** Transport of drugs into microwells with time: (i) 200 s, (ii) 800 s. Mass transport simulation was performed by considering arbitrary 1% (v/v) concentration of species from an inlet

The device consists of two layers: (1) bottom layer (overall size: 48.2 × 43.8 × 1.4 mm), (2) top layer (overall size: 47.2 × 42.8 × 5.5 mm). The bottom layer contains a channel network with a set of serpentine loops to mix the drug combinations. It also incorporates an array of seven microwells at the end of each serpentine loop to form spheroids (Fig. 1A). Top layer contains fluid reservoirs with 35 µl capacity and inlet and outlet holes (Fig. 1B). The overall volume of the microfluidic channel network is 45 µl.

All channels have a rectangular cross-section of 400 × 200 µm. Cylindrical microwells are 400 µm in diameter with 400 µm in depth. The bottom layer also contains 10 milliwells which are 1 mm in diameter with a depth of 600 µm. These milliwells capture loose cells and aggregates from the channels after cell seeding, thus facilitating formation of uniform-sized, single spheroids in each microwell. Fluid reservoirs on the top layer are connected to these 10 millwells through 1.5 mm punched holes, each having a depth of 3.5 mm. Fluid reservoirs prevent the cell culture medium from drying out in the bottom layer and facilitate fluid exchange without bubble formation in channels.

The microfluidic design consists of three inlet ports (IP) for the perfusion of three different drugs, as shown in yellow, red, and blue colors (Fig. 1A). Three serpentine loops have been set up at 0°, 90°, and 180° for this purpose. Moreover, another three serpentine loops set up at 45°, 135°, and 270° allow testing of three drug pairs, as shown by orange, purple, and green colors. A serpentine loop in the center with a grey color tests a combination of all three drugs. Drug combinations exit from the device through four outlet ports (OP). Removable ports (RP) serve as outlets during cell seeding and can be sealed during drug perfusion experiments using 3D-printed inserts. RP also serves to remove air bubbles from the channels during the experiment. Each drug combination can be tested on a set of seven spheroids. Hence, forty-nine microwells have been arranged for seven drug combinations. A method devised for forming uniform-sized, single spheroids in each microwell has been described in detail in the supplementary material (Figure S4).

The design of the device was validated with computational simulations (COMSOL Multiphysics). Firstly, the serpentine loop length was decided based on mixing (%). Mixing (%) and concentration of species were measured at the end of the serpentine loop along a 300 µm vertical line. We obtained average mixing (%) of 98.3% at 2 µl/min outflow rate in a serpentine loop with a length of 57 mm (Fig. 1C). Hence serpentine loop length of 57 mm was decided. Once the length of the serpentine loop was finalized, velocity simulations were carried out in the designed serpentine loop network. Velocity distribution in outlet cell culture and inlet cell culture areas was slightly different, with the maximum velocity (*V*_*max*_) in the outlet cell culture area = 0.4231 mm/s and inlet cell culture area = 0.561 mm/s (Fig. 1D) (cell culture area is shown in green dotted box). Moreover, deeper areas of microwells remain devoid of fluid flow, thereby protecting spheroids from shear stress due to dynamic perfusion (Fig. 1E). However, drugs penetrated 400 µm deep microwells thoroughly within 800 s at a flow rate of 2 µl/min with convection and diffusion transport mechanisms (Fig. 1F). Hence, with a designed microwell depth of 400 µm, we can protect the spheroids from shear stress, while treating them with the drugs dynamically.

### Comparison of photocurable resins

Three key properties are crucial for microfluidic devices fabricated using 3D printing [21]: (1) low surface roughness, which enhances plasma bonding strength (Figure S5A–E) (2) high optical transparency, essential for brightfield imaging of cells (Figure S5F), and (3) high dimensional accuracy, vital for forming uniform sized spheroids and ensuring accurate drug mixing as per the simulation results (Figure S6-S8). Therefore, we assessed these three properties in PDMS devices derived from soft-lithography molds made from well-known commercial photocurable resins. Lastly, we focused on the dimensional accuracy of seven microwell arrays (Figure S7B). As the device contains these arrays at seven different locations, we suspected that the dimensional variation in any of these arrays could disturb the uniform size distribution of spheroids. Hence, we hypothesized that a low standard deviation in measured microwell sizes would facilitate the formation of uniform-sized spheroids or spheroids. Based on the extensive comparison study, we selected standard clear resin to fabricate the soft-lithography molds for PDMS based spheroids-on-a-chip. Further details can be found in supplementary material.

### Device characterization and validation with tumor cell line

PDMS spheroids-on-a-chip fabricated from standard clear resin molds were used to measure mixing (%). We achieved experimental mixing (%) of 96.54 ± 0.063%, which closely matched the average mixing (%) of 98.3% obtained from simulation results. The detailed mixing (%) results can be found in the supplementary material (Figure S9A–C).

3D printing introduced geometrical variations in the fabricated device, as shown in Figure S7. These variations were not considered in simulation results in Section "Microfluidic device design and computational validation" since the main purpose behind computational simulations was to optimize the design and serpentine loop length before choosing the manufacturing process and material. However, we have performed the mixing simulation by considering geometrical variations introduced by standard clear resin-based fabrication strategy. We found that mixing results were in close agreement with the reported results in Section "Microfluidic device design and computational validation" and experimental mixing (data not shown).

Furthermore, we performed surface characterization of the PDMS device after applying 2% (w/v) coating of pluronic F127 for 24 h, intended to induce cell aggregation using XPS and contact angle goniometry. We found minor peak showing presence of CH_2_CH_2_O group on PDMS due to pluronic F127 coating at 286.6 eV. In line with C1s spectra, the survey scan showed an increase in the amount of atomic carbon and oxygen due to presence of pluronic groups. Moreover, the presence of pluronic groups rendered PDMS surface hydrophilic (~ 56°), whereas native PDMS remained hydrophobic (~ 95). The detailed results are shown in supplementary material (Figure S10B-C).

A breast cancer cell line (MCF-7) was cultured in the PDMS device to assess biocompatibility, uniform-sized spheroid formation with the developed cell seeding method (Figure S4), standard clear resin mold-based fabrication strategy (Section "Comparison of photocurable resins"), and pluronic F127 based coating strategy. The breast cancer cell line was used only to evaluate the above mentioned details and not to perform any drug screening as the primary focus of the manuscript is on primary cells and personalized drug testing. We found that uniform-sized tumor spheroids could be formed with the standard-clear resin mold-based fabrication strategy with the maximum standard deviation of 11.97 µm found on day 6. We had hypothesized that a low standard deviation in measured microwell sizes would enable formation of uniform-sized spheroids in PDMS device fabricated from standard clear resin mold (Section "Comparison of photocurable resins"). Our MCF 7 spheroid size results corroborate this hypothesis. Additionally, MCF-7 cell line spheroids remained highly viable for 6 days in the chip, proving the device's excellent biocompatibility. The detailed results are described in the supplementary section (Figure S10D-H). Next, we isolated primary oral stem-like cancer cells and cultured them in the device.

### Primary oral tumor stem-like cells isolation, characterization, and culture in the device

- Primary oral stem-like cells characterization:

Isolated cells formed primary and secondary orospheres proving the self-renewal capacity of stem-like cells (Figure S11A) [37]. Figure S11B shows aldehyde dehydrogenase (ALDH1) positive expression, indicating stem-like cells presence [38]. Positive expression of epithelial cell adhesion molecule (EpCAM) (Figure S11C) and negative expression of alpha smooth muscle actin (αSMA) (Figure S11D) represent the absence of the stromal cells in isolated enriched oral stem-like cells. Moreover, flow cytometry analysis with CD 44^high^/CD 24^low^ expression also corroborated the presence of cancer stem-like cells in all three patient-derived cell population (Figure S11E) [39]. Furthermore, CD 44 expressing cells in patient 1 (91%) were higher than patient 2 (84.3%) and patient 3 (86%). The absence of CD antigens associated with hematopoietic and immune cells confirmed the purity of isolated cells. Next, we cultured primary cells in the developed microfluidic device.

**Fig. 2:**
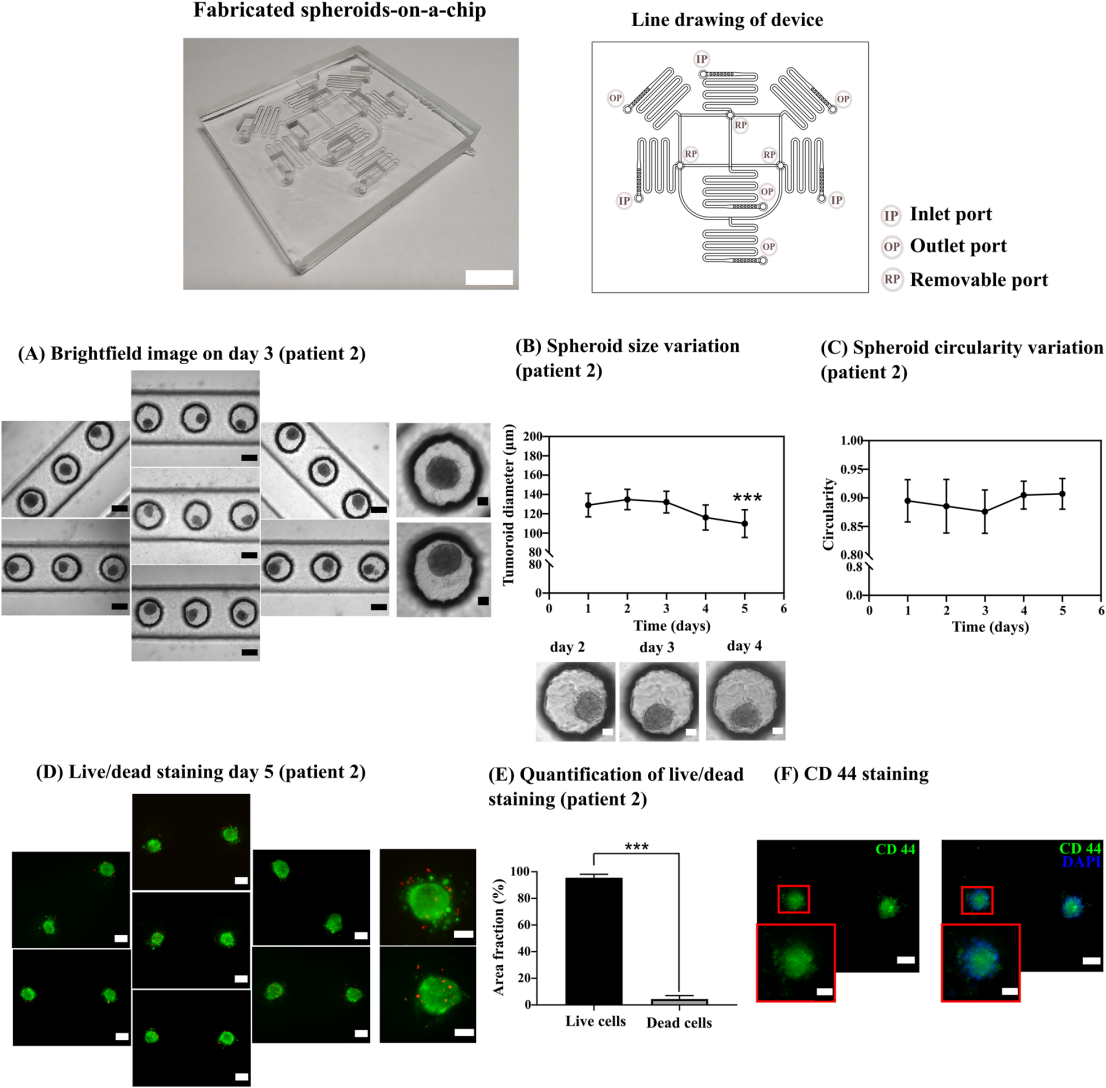
**A** Patient 2 derived primary oral stem-like tumor cells are seeded with 12 µl/min flow rate. Brightfield images of spheroids were captured on day 3 (left panel scale bar = 200 µm and right magnified panel scale bar = 50 µm). **B** Spheroid size variation with time measured in ImageJ (scale bar = 50 µm). (Mean ± SD, Student’s t-test, *p* < 0.001 (***) with day 1, *n* = 49 spheroids) (**C**) Spheroid circularity with time measured in ImageJ (Mean ± SD, *n* = 49 spheroids). **D** Live/dead staining on day 5 showing live cells in green color and dead cells in red color (scale bar in left panel = 100 µm and right magnified panel = 50 µm). **E** Quantification of live/dead staining on day 5. (Mean ± SD, Student’s t-test, *p* < 0.001 (***), *n* = 49 spheroids). **F** Immunofluorescence with CD 44 marker, showing green cells positive for CD 44 and blue color showing DAPI staining (scale bar = 100 µm and magnified image scale bar = 50 µm). The spheroids cultured in left-most and right-most microwell arrays have been represented in the horizontal orientation

- Oral spheroids culture in device:

Figure S12 shows the H&E staining of biopsy samples derived from oral tumor patients, corresponding well with their respective diagnoses (Table 2). Figure 2A shows a brightfield image of stem-like spheroids derived from patient 2 cultured in the chip on day 3. The size of spheroids was significantly lower on day 5 than day 1, suggesting compaction with time. Besides, the mean spheroid size remained below 150 µm during 5 days with a maximum standard deviation of 14.35 µm on day 5 (Fig. 2B). Primary spheroids maintained a mean circularity index close to 0.9 during culture (Fig. 2C). In addition, they maintained excellent viability of more than 90% during in vitro culture on chip (Fig. 2D, E). We also found that CD44, a major cancer stem cell marker, was highly expressed in the cultured spheroids (Fig. 2F). The results presented in this section demonstrate that the developed device is capable of forming uniform-sized primary stem-like spheroids, maintaining them with high viability, and preserving their stem-like properties.

### Personalized combinatorial drug screening

We isolated oral tumor stem-like cells from biopsy samples of three patients and cultured them in the developed spheroids-on-a-chip for combinatorial drug testing. We selected well-known oral cancer chemotherapy regimen, including paclitaxel (1 µM) (PTX), 5 Fluorouracil (20 µM) (5-Fu), and cisplatin (5 µM) (CDDP). In each of the three serpentine loops, the pairwise mixing reduces the concentration of each drug to approximately half. Likewise, in the central loop, the concentration of each drug is reduced to about one-third. This estimation is based on the mixing characterization results, which showed 96.54 ± 0.063% experimental mixing (%) in serpentine loops (Figure S9B).

**Fig. 3:**
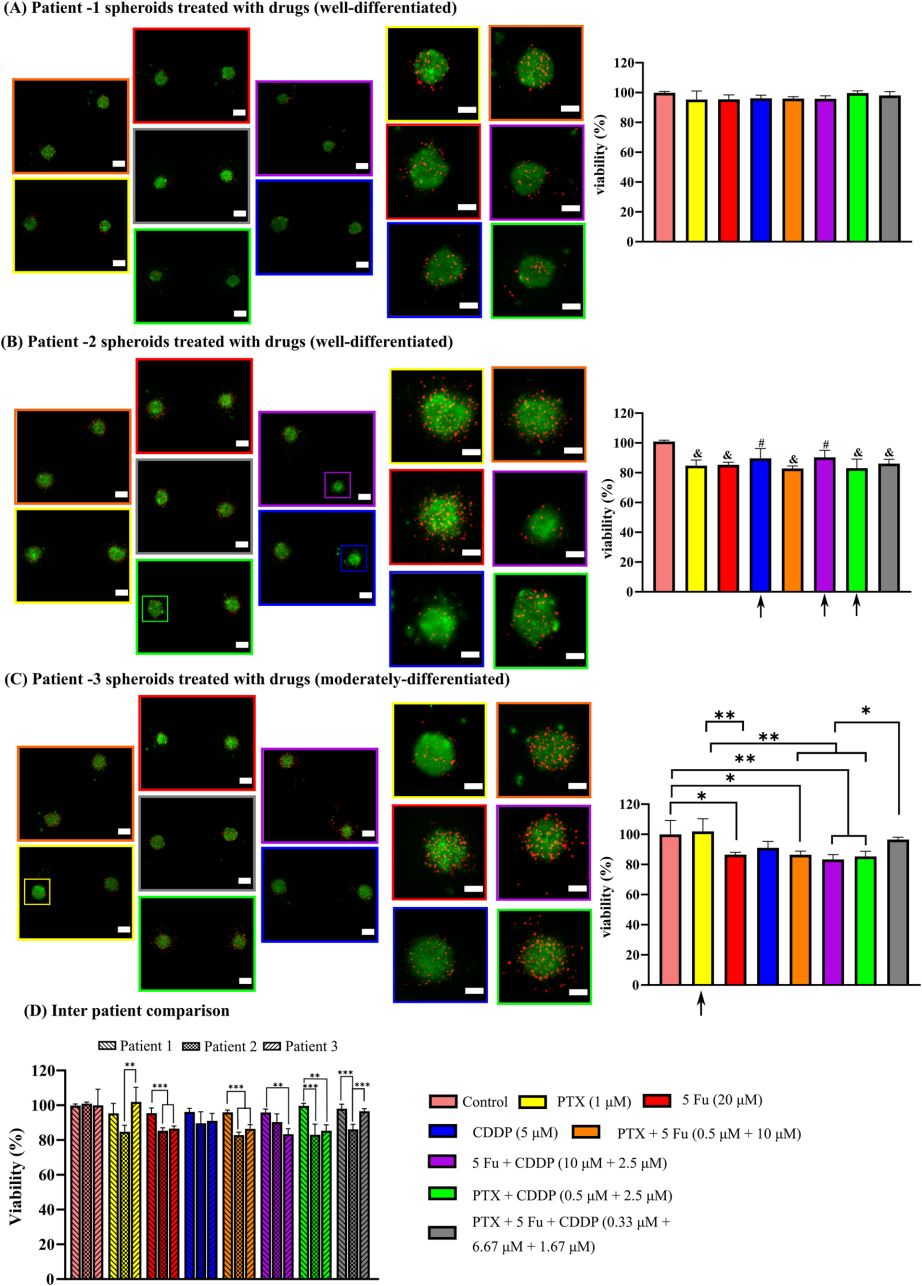
Live/dead staining after drug perfusion at 2 µl/min for 6 h and 2 h static incubation on day 3: **A** Patient 1 spheroids showing live cells in green and dead cells in red color. **B** Patient 2 spheroids showing live cells in green and dead cells in red color. **C** Patient 3 spheroids showing live cells in green and dead cells in red color. **D** Inter-patient comparison. (Reduced chemosensitivity in a few spheroids in patient 2 and 3 has been shown by small colored squares in the fluorescent images and arrows showing high standard deviation in the bar graph). (All data normalized to control, data presented as Mean ± SD, A one-way ANOVA, Tukey’s post hoc test, *n* = 7 spheroids measured for each treatment group, ‘&’ shows *p* < 0.001 with control, ‘#’ shows *p* < 0.05 with control) The spheroids cultured in left-most and right-most microwell arrays have been shown in the horizontal orientation. Controls were treated with 0.1% DMSO. Scale bars in left panels are 100 µm and right magnified panels are 50 µm

- Patient 1:

Patient 1 spheroids were highly resistant to all the seven drug combinations in the device (Fig. 3A). We found no significant difference between various drug groups and a control.

- Patient 2:

All chemotherapy combinations produced significantly higher cell death compared to the control (Fig. 3B). Although cisplatin monotherapy was significantly different from the control, few spheroids in the cisplatin microwell array showed reduced sensitivity to it (shown by small colored square in the image and arrow showing high standard deviation in the bar graph). Microwell array showing spheroids with reduced cisplatin sensitivity can be also observed from Figure S13A. Furthermore, several spheroids treated with pair-wise combinations of cisplatin + 5 Fu (Figure S13B) and cisplatin + paclitaxel (Figure S13C) also showed reduced sensitivity. This might be indicating cisplatin drug resistance in patient 2 derived spheroids. As there is no significant difference among the individual drug groups, treatment with paclitaxel + 5 Fu and all three drugs (paclitaxel + 5 Fu + cisplatin) possibly indicate additive effect of drugs in combination (combination index = 1) [36]. It can be concluded that for treatment of patient 2, a therapy involving the combination of all three drugs, or a pairwise combination of paclitaxel and 5-Fu, or even monotherapies using either paclitaxel or 5-Fu, may prove beneficial.

- Patient 3:

Spheroids derived from patient 3 didn’t respond well to paclitaxel monotherapy. Few of the spheroids seemed completely non-responsive (shown by small colored square in the image and arrow showing high standard deviation in the bar graph) (Figure S13D & Fig. 3C). Moreover, treatment with all three drugs was ineffective. Patient 3 spheroids also demonstrated cisplatin resistance, which was observed in a few spheroids in patient 2. Pair-wise combination of paclitaxel with either cisplatin or 5 Fu produced higher cytotoxicity than mono-drug therapy of paclitaxel. Hence, these two combinations possibly indicate the synergistic effect of drugs (combination index < 1) [36]. Besides, the pair-wise combination of cisplatin + 5 Fu represents additive effect of drugs (combination index = 1). Tumor response to 5 Fu monodrug therapy was better than paclitaxel. Hence, 5 Fu mono drug therapy or one of the three pair-wise combinations can be effective for patient 3. Overall, the responses to various drug combinations varied significantly among spheroids derived from all three patients. These responses indicate inter-patient heterogeneity captured at clinically relevant dosages (Fig. 3D).

### Evaluation of morphology of oral spheroids

**Fig. 4:**
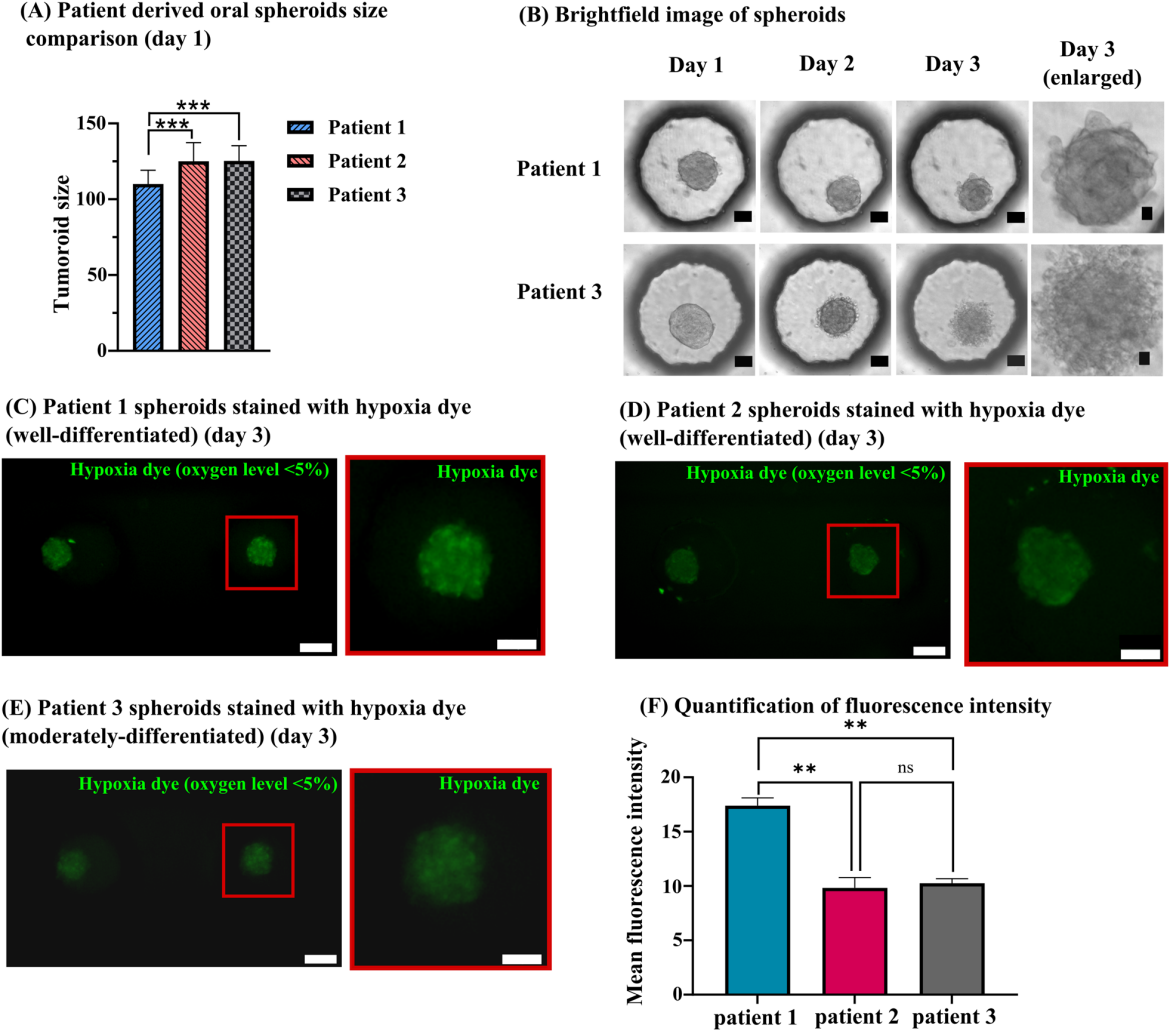
**A** Size comparison of patient-derived spheroids on day 1 (Mean ± SD, A one-way ANOVA, Tukey’s post hoc test, *p* < 0.001 (***), *n* = 49 spheroids). **B** Brightfield images showing morphological changes in patient 1 and patient 3 spheroids from day 1 to day 3 (scale bar = 50 µm and enlarged image scale bar = 10 µm). **C** Image iT- green hypoxia reagent expression in patient 1 spheroids on day 3 (the dye detects oxygen level < 5% and its intensity is inversely proportional to the oxygen concentration). **D** Hypoxia dye expression in patient 2 spheroids (day 3) (**E**) Hypoxia dye expression in patient 3 spheroids (day 3). **F** Quantification of fluorescence intensity of hypoxia dye (data presented as mean ± SEM, A one-way ANOVA, Tukey’s post hoc test, *p* < 0.01 (**), *n* = 3 spheroids). **C**–**E**: All scale bars in left images are 100 µm and right magnified images are 50 µm

Figure 4A shows significantly lower mean size of patient 1 derived spheroids (110 µm) than patient 2 and 3 derived spheroids (125 µm) on day 1 with the same cell seeding number (6000 cells/µl). Figure 4B depicts the more compact size of patient 1 derived spheroids than patient 3 derived spheroids on day 1. As shown in day 3 images, patient 1 derived spheroids maintained tight cellular junctions, whereas the morphology of patient 3 derived spheroids suggested reduced cell–cell contact, especially in the boundary region. Unlike patient 1 spheroids, a clear boundary was not visible in patient 3 spheroids. Patient 2 derived spheroids also maintained relatively tighter cellular boundary than patient 3 spheroids on day 3 (Fig. 2A). In conclusion, well-differentiated spheroids from patient 1 and patient 2 demonstrated relatively tighter cellular junctions than moderately differentiated patient 3 derived spheroids.

### Evaluation of oxygen levels in spheroids

The oxygen permeability in PDMS is dependent on its thickness [40, 41]. As shown by Lee et al*.*, the oxygen permeability significantly decreases when thickness of bottom PDMS block exceeds 1 mm [40]. As the bottom PDMS block in our microfluidic device has thickness of 1.4 mm, we decided to evaluate the oxygen levels in cultured spheroids.

We utilized hypoxia dye to detect oxygen levels in cultured oral spheroids. Image iT-green hypoxia reagent detects oxygen levels less than 5% in cells, and its intensity is inversely proportional to the oxygen levels. We found all three patients-derived spheroids stained positively, exhibiting oxygen levels less than 5% (Fig. 4C-E). However, patient 1 spheroids possessed brighter expression of the dye, indicating lower levels of oxygen than patient 2 and 3 spheroids (Fig. 4F).

## Discussion

In the present study, we developed a personalized oral stem-like spheroids-on-a-chip system. This system successfully captured inter-patient tumor heterogeneity, identified the most effective drug combinations, therapy resistances, and revealed patient-specific differences in spheroid morphology, size, and oxygen levels.

- Clinical correlation of spheroid morphology and size:

Patient 1 and patient 2 were diagnosed with well-differentiated, and patient 3 was diagnosed with moderately differentiated oral squamous cell carcinoma. Moderately and poorly differentiated tumors are known to be more aggressive and invasive than well-differentiated tumors [42]. Moreover, moderately differentiated tumors are also associated with lower E-cadherin expression levels than well-differentiated tumors [43, 44]. Low E-cadherin expression leads to reduced cell–cell contact, especially in boundary region in head and neck cancer spheroids derived from FaDu, Hep2, and Hep2-Tax [45]. In line with their results, we found patient 3 derived moderately differentiated spheroids possessed reduced cell–cell contact in boundary region and patient 1 and patient 2 derived well-differentiated spheroids maintained tight cellular junctions (Figs. 4B and 2A). Moreover, a similar observation was found in previous pancreatic cancer study [46]. The Capan-1 cell line, derived from well-differentiated cancer, exhibited a discernible tight boundary, in contrast to the BxPc-3 cell line spheroids, which originated from moderately differentiated tumors and displayed loosely adhered cells in their boundary regions [46].

The notably smaller average size of spheroids derived from patient 1 implies a greater accumulation of E-cadherin in them compared to those from patients 2 and 3 [45]. Consequently, this higher E-cadherin level may lead to proliferation suppression, accounting for their reduced size [47]. However, further validation of this hypothesis will be necessary through flow cytometry analysis to assess the E-cadherin expression in the spheroids.

- Analysis of differential oxygen levels in spheroids:

All three patients-derived spheroids expressed regions with oxygen levels less than 5% (Fig. 4C-F). Patient 1 spheroids were found with the brightest expression of hypoxia dye. Apart from gas permeability limitations of PDMS, we believe that the compact size and elevated E-cadherin accumulation in patient 1 spheroids might have attenuated intracellular space for oxygen diffusion, resulting in the lowest oxygen concentration [48, 49].

Moreover, dissimilar patient-specific hypoxia dye expression was observed, which was not specifically limited to the central core region of the spheroid. This observation was in line with the previous study showing dissimilar hypoxia dye expression in four head and neck cancer cell lines that were derived from different patients [50]. The study also demonstrated OSC-19 spheroids with evenly distributed hypoxia dye expression throughout the spheroid [50].

Normal tissues generally possess 5% oxygen concentration (physoxia) [50, 51]. However, it has been found that the median oxygen level in cancers is much lower (≤ 4.2% O_2_). Solid head and neck tumors typically exhibit physiological hypoxia with oxygen levels ranging from 1–2% O_2_ [50, 51]. This indicates that our device successfully sustained spheroids at oxygen levels relevant to solid head and neck tumors, without adversely affecting their viability during 5 days (Fig. 2E).

The presence of cancer stem cells and hypoxic regions in solid tumors are associated with poor prognosis and treatment outcomes. Hence, the developed model is suitable to test potential drug combinations effective in hypoxic regions and eradicating cancer stem cell population.

- Analysis of heterogeneous drug response and clinical correlation:

Finally, we used a well-known oral cancer chemotherapy regimen, including paclitaxel, 5 Fu, and cisplatin for FDT. We found that patient 1 spheroids possessed the highest resistance to various drug combinations, whereas spheroids derived from patients 2 and 3 were responsive to the same.

The observed highest drug resistance in patient 1 spheroids can be explained by the highest hypoxia dye expression found in them (Fig. 4F). Hypoxia can be one of the reasons behind dormant cell fractions in head & neck cancers [52]. As the chemotherapeutic drugs act on rapidly dividing cells, dormant or slowly dividing cell fractions in hypoxic regions might not respond to the same [19, 20]. Greater hypoxia dye expression in patient 1 derived spheroids than patient 2 and 3 spheroids suggested relatively lower proliferation rate of cells in them, explaining the drug resistance exhibited by spheroids from patient 1. Besides, as stated before, lower proliferation due to the higher E-cadherin accumulation in patient 1 derived spheroids also explains the higher drug resistance.

Lower sensitivity to cisplatin and its combinations remains a challenge in head & neck cancer chemotherapy [53]. Previous studies identified a set of genes that predicted cisplatin resistance in head & neck cancer patients [53, 54]. We believe that patient-specific gene expression studies will be required to confirm the exact mechanism behind the cisplatin resistance captured in this study.

Furthermore, few spheroids with reduced sensitivity to cisplatin and its combinations (patient 2), among other responsive spheroids might also indicate intra-patient heterogeneity. Intra-patient heterogeneity presents a critical challenge in functional drug testing based on microfluidic devices, as reported by the previous studies [55, 56].

Emerging literature is revealing the coexistence of two populations of cancer stem cells: (1) EMT: mesenchymal type and (2) non-EMT: epithelial type. These two populations give rise to phenotypic diversity at intra patient level in patient-derived oral tumors [57, 58]. Moreover, the orosphere-based approach used for cancer stem cells (CSC) enrichment is also implicated in producing diversity within the CSC population, as proposed by Xie et al. [58]

We had selected dynamic drug treatment time of 6 h. The slope of typical concentration vs time curve for 24 h chemotherapeutic drug infusion was assumed to be constant near the peak plasma level for a short period of 6 h [28, 33]. Therefore, we believe that drug perfusion at constant *C*_*max*_ for 6 h reliably replicates the effects of the drugs, as experienced by native tumor. A longer infusion time may produce greater cytotoxicity, but it will significantly exaggerate the effects of drugs, leading to misinterpretation of drug responses. However, future efforts should be directed towards exposing spheroids to the entire pharmacokinetic profile and repeated dosing to reach IC_50_.

PDMS is known to adsorb small molecules such as drugs [59]. However, the introduction of polyethylene glycol (PEG) moiety on PDMS minimizes the drug adsorption, as shown by the recent study [60]. Hence, we have coated widely used PEG containing tri-block copolymer pluronic F127 on PDMS to prevent such non-specific adsorption [11, 61]. Usually, such triblock copolymers also prevent cell adhesion and allow spheroid formation by polyethylene glycol groups, that are known to prevent protein adsorption by steric repulsion [62, 63]. However, we acknowledge the fact that complete drug adsorption prevention may not be possible with the existing PEG based strategies. Therefore, other polymers such as polymethyl methacrylate (PMMA), polystyrene (PS), polycarbonate (PC), cyclic olefin copolymer (COC) can be explored as a microfluidic materials.

- Comparison of the platform with the existing literature:

There are several platforms based on 3D spheroids and drug screening reported in the literature [64, 65]. Similarly, there are several platforms reported for combinatorial drug screening [66, 67]. However, majority of the previously reported platforms used immortal cancer cell lines or PDX cell lines. As per Table S2, very few studies from the last 6 years used primary cells isolated from the fresh patient derived tissue. We could also substantiate our claim based on the recent Bouquerel et al. systematic review paper on tumor-on-chip covering last 15 years with over 300 publications [13]. According to their data, only 5% of the papers used isolated cells from the fresh patient-derived tumor samples. The majority of the studies (70%) used immortal cancer cell lines. Although, cancer cell lines offer good reproducibility, extensive passaging capacity, and high proliferation capacity, it is also well-known and accepted that cell lines do not completely represent relevant models of in vivo tumors, since indeterminate transcriptomic, epigenetic, genetic and phenotype changes may occur during cell immortalization [13]. Further, they also do not recapitulate the inter-patient heterogeneity, which is essential for personalized functional drug testing and is captured in our study using primary cancer cells.

Personalized drug screening studies focusing on oral cancer have been rare. Majority of the studies focused on breast, lung, colorectal and pancreatic cancers in the last 15 years [13]. Additionally, papers focusing on cancer progenitor population (cancer stem cells), which is known to be chemoresistant and a reason behind cancer relapse, are also rare in spheroids-on-chips.

Additionally, from Table S2, only one study reported hypoxia levels in the cultured spheroids. Hypoxia emerges as a pivotal factor in tumor progression and treatment resistance, through a response mainly ascribed to hypoxia-inducible factors (HIFs) [13]. The highest drug resistance observed in patient 1 derived spheroids was correlated with the highest hypoxia dye expression in patient 1 spheroids, demonstrating the importance of maintaining in vivo like oxygen levels in in vitro cultures.

As shown in Table S2, none of the studies reported correlation of drug screening results with patient’s clinical histopathological data. Additionally, differential spheroid morphologies according to the patient’s tumor differentiation status were also not observed in any of the papers. Hence, this differentiates our paper from the previous papers published in this area.

Finally, very few papers reported the use of 3D printing for fabricating the tumor-on-chips. Additionally, none of the previous papers have compared 3D printing materials for fabricating spheroids-on-chip with the same depth and extent as our study.

The morphological differences representing the patient’s tumor differentiation status observed under dynamic flow and 5% O_2_ levels were absent in static well-plate-based cultures [34]. Due to hypoxic conditions and compactness, patient 1 derived spheroids were resistant to drug treatments on microfluidic platform, in contrast to the outcomes observed on static well-plate cultures [34]. Hence, microfluidic platforms have the potential to offer better insights into patient-derived samples than conventional well-plate-based cultures.

Several limitations of the work involve lack of functional vasculature, lack of other cell types and lack of molecular studies. Vasculature is an integral part of the tumor microenvironment and helps to capture actual drug exposures to the tumor spheroids in vitro. Additionally, testing of anti-angiogenic therapy is also possible with in vitro vasculature [68]. We will explore this as a part of future work with co-culturing endothelial cells.

As per Table S2, in situ imaging has been the most-prominent technique for qualitative and quantitative characterization of spheroids-on-chips. Additionally, inadequate cell number from one chip and inaccessibility of the spheroids from the chip were the two main constraints for the lack of molecular studies in this paper. To address this limitation, we will increase the number of microwells and introduce a higher number of cells to obtain adequate amount of RNA in a modified design. Further, we are also currently optimizing the spheroids retrieval from the chip with high flow rate of 500–1000 µl/min.

As per Table S2, only one study incorporated more than three patient samples in the personalized medicine study [69]. Hence, we believe that a sample size of three patients is adequate to demonstrate the proof-of-concept of our platform. However, our future studies will include a greater number of patient samples to justify clinical utility of the platform. Additionally, we will treat the spheroids with the same drugs being administered to the patient to correlate the device data directly to clinical outcomes.

CD 44 is one of the most important and widely reported CSC markers in head & neck cancer, as confirmed by the previous studies incorporating patient data [70–72]. As shown in Fig. 2F, the grown spheroids in microfluidic device demonstrated high positive expression of CD 44, confirming the maintenance of stem cell-like properties. However, lack of other markers such as Oct-4, Sox2, Nanog, and SSEA-1 is a limitation of the study.

The tumor microenvironment consists of other cell types, such as fibroblasts, immune cells, and endothelial cells. Moreover, the stromal cells significantly affect the drug response [73]. We believe that co-culture of stromal cells and tumor cells in the developed device will capture the complexity of tumors more accurately in the future studies.

## Conclusion

In this study, we have reported a novel 3D printing-based device capable of capturing inter-patient tumor heterogeneity, identifying the most effective drug combinations, therapy resistances and revealing patient-specific differences in spheroid morphology, size, and oxygen levels. These characteristics also correlated with each patient’s diagnosis from clinical histopathological report. The oral spheroids cultured in the microfluidic system expressed the cancer stemness marker (CD44^+^) and maintained high viability for 5 days. The highest drug resistance captured in patient 1 derived spheroids correlated with the lowest oxygen levels in them. Interestingly, few of the drug combinations produced additive and synergistic effects on microfluidic chip. To the best of our knowledge, this is the first report demonstrating extensive work on development of microfluidic based oral cancer spheroid model for personalized combinatorial drug screening. Furthermore, the obtained clinical correlation of drug screening data represents a significant advancement over previously reported personalized spheroid-based microfluidic devices. Finally, the maintenance of patient-derived spheroids with high viability under oral cancer relevant oxygen levels of less than 5% O_2_ is a more realistic representation of solid tumor microenvironment in our developed device.

### Supplementary Information

Supplementary Material 1.

## Data Availability

The datasets used and/or analysed during the current study are available from the corresponding author on reasonable request.

## Abbreviations

GLOBOCAN: Global cancer observatory
ICMR: Indian council of medical research
FDT: Functional drug testing
FDM: Fused deposition modeling
CSC: Cancer stem cells
PDMS: Polydimethylsiloxane
PI: Propidium iodide
PTX: Paclitaxel PTX
5 Fu: 5 Fluorouracil
CDDP: Cisplatin
CI: Combination index
IP: Inlet ports
OP: Outlet ports
RP: Removable ports
ALDH1: Aldehyde dehydrogenase
EpCAM: Epithelial cell adhesion molecule
αSMA: Alpha smooth muscle actin
PEG: Polyethylene glycol
PMMA: Polymethyl methacrylate
PS: Polystyrene
PC: Polycarbonate
COC: Cyclic olefin copolymer
