# Supplementary material for: 3D stem-like spheroids-on-a-chip for personalized combinatorial drug testing in oral cancer
Source: J Nanobiotechnology. 2024 Jun 18;22:344. doi: 10.1186/s12951-024-02625-y (PMC11186147; doi:10.1186/s12951-024-02625-y)
Supplement: Supplementary file 1 — Supplementary Material 1. [file 12951_2024_2625_MOESM1_ESM.pdf]

Supplementary material

3D stem-like spheroids-on-a-chip for personalized combinatorial drug testing in oral cancer

Viraj Mehta[#a], Sukanya Vilikkathala Sudhakaran[#a], Vijaykumar Nellore[a], Srinivas Madduri[b], Subha Narayan Rath[a]*

[a] Regenerative Medicine and Stem cell Laboratory (RMS), Department of Biomedical Engineering, Indian Institute of Technology Hyderabad, Kandi, Telangana, India

[b] Department of Surgery, University of Geneva, 1205 Geneva, Switzerland

* **Correspondence to:**

Department of Biomedical Engineering,

Indian Institute of Technology Hyderabad (IITH),

Kandi, Sangareddy, 502285, Telangana, India.

E-mail address: subharath@bme.iith.ac.in (S.N. Rath)

Phone: +91 40 2301 6103

[#]Equal contribution

1. Supplementary materials and methods

1.1 Comparison of resins

Table S1 lists the 3D printing apparatus, type of technology, horizontal resolution, and layer height for each resin compared in the present study.

Table S1. List of resins for comparative study

3D printing apparatus	Type of technology	XY Resolution	Resin	Layer height
3D Systems ProJet 6000 HD	Stereolithography (SLA)	4000 DPI (6.35 µm)	Accura 25, Accura ClearVue	Accura 25 (100 µm), Accura ClearVue (50 µm)
3D Systems FabPro 1000	Digital light processing (DLP)	65 µm	Proto GRY	50 µm
3D Systems ProJet MJP 2500	MultiJet printing (MJP)	1600 × 900 DPI (15.8 × 28.2 µm)	VisiJet M2R-CL	32 µm
Formlabs Form 3B	Low force stereolithography (LFS)	25 µm	Standard Clear	25 µm

A mold was designed with rectangular channels of depth 200 µm and width varying from 50 to 500 µm. Moreover, it also contained cylindrical microwells with a depth of 400 µm and a diameter varying from 50 to 500 µm. Next, PDMS was cured over each device, and the cross-sections of features were measured using a light microscope (EVOS M7000 optical microscope, Thermofisher Scientific, Waltham, MA, USA). We plotted percentage dimensional deviation calculated by **equation S1**, as mentioned below.

$$Dimensional\ deviation(\ \%\ ) = \frac{|\ designed\ dimension - measured\ dimension|}{designed\ dimension} \times 100 \qquad \text{(S1)}$$

Optical transmittance was measured according to the previous protocol [1]. Briefly, PDMS was punched to fit in a 96-well plate, and absorbance was measured using a microplate

reader (Enspire multimode plate reader, PerkinElmer, MA, USA) between 350-750 nm. Absorbance values were converted to transmittance and plotted at 550 nm. For roughness measurement, 470 × 350 μm area was scanned with 20x magnification and vertical step size of 0.1-0.2 μm (Zeta profilometer, KLA-Tencor, USA). Finally, arithmetical mean height (S_a) and root-mean-square height (S_q) were obtained.

1.2 Fabrication procedure

Soft-lithography molds for each microfluidic device layer were modeled in Autodesk Inventor 2019 student edition. The modeled files were exported in stereolithography (*.stl*) file format. Bottom layer molds were printed in Formlabs standard clear resin in Formlabs Form 3B SLA apparatus (iTic foundation, IIT Hyderabad) with a layer height of 25 μm (**Figure S1(A)**). Top layer molds were printed from Accura 25 in 3D systems ProJet 7000 HD (3D print India, Mumbai, India) in 100 μm layer height. The molds were printed parallel to the build plate for better surface finish and accuracy. 3D printed inserts for sealing removable ports were printed from BioMed Clear biocompatible resin in Formlabs Form 3B printer. After printing, clear resin molds were washed with isopropyl alcohol (IPA) for 20 min and cured at 60°C for 30 min. Next, the PDMS base and curing agent were mixed at a ratio of 10:1 (w/w), degassed, and poured over the mold surface. Subsequently, the molds were heated at 60°C for 3-4 h for PDMS solidification (Figure S1(A)). Cured PDMS devices were treated with air plasma (Harrick Plasma, Ithaca NY, USA) at 1000 mTorr and 30 W for 1 min 15 sec (**Figure S1(B))**. Treated devices were bonded and heated at 100°C for 42 h for hydrophobicity restoration. Finally, they were coated with 2% (w/v) pluronic F127 solution for 24 h before cell seeding (**Figure S1(C)).**

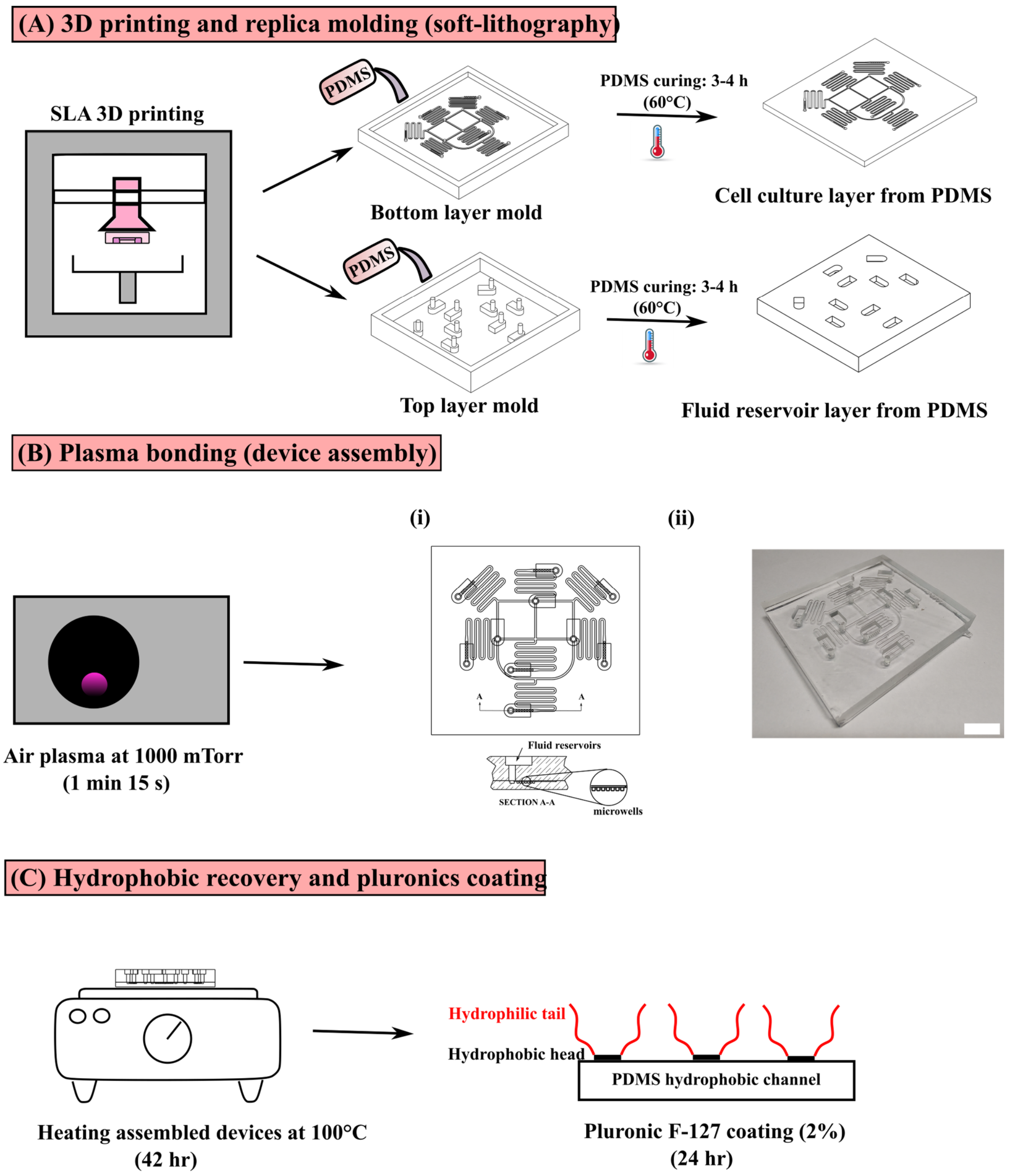

Figure S1. (A) Top and bottom layer molds are 3D printed, which is followed by the casting of PDMS. Next, PDMS is cured at 60°C for 3-4 h. (B) Both PDMS blocks are bonded using air plasma treatment at 1000 mTorr for 1 min 15 s: (i) Cross-sectional view showing microwells, (ii) Assembled PDMS device used for drug screening (scale bar = 10 mm). (C) The assembled device is heated at 100°C for hydrophobic recovery and subsequently coated with pluronic F127 (2% w/v) for 24 h to prevent cell adhesion and facilitate spheroid formation.

1.3 Mixing characterization

While direct drugs weren't employed for mixing characterization, food color dyes with molecular weights similar to those of drugs were tested in the current study. Blue dye (1.3 % v/v) containing brilliant blue (molecular weight 792.9 g/mol) and yellow dye (2.7 % v/v) containing tartrazine (molecular weight 534.36 g/mol) were prepared in deionized water. Blue dye solution (representing drug A), deionized water (representing drug B), and yellow dye solution (representing drug C) were withdrawn from three inlets towards four outlet ports by applying a negative pressure by the syringe pump at 2 µl/min (Chemyx Inc., Stafford, TX, USA). Brightfield images of all serpentine loops were captured. Mixing quantification of blue dye and water in one of the serpentine loops was performed in ImageJ. Firstly, images were covered in HSB (hue, saturation, brightness) stack, and a saturation channel was selected. Next, gray values were plotted along a 300 µm vertical line at the end of the serpentine loop. The mean gray value of the externally mixed blue color solution was measured by perfusing it in the device. Mixing (%) was calculated according to **equation S2**.

$$mixing\ (\ \%\ )\ =\frac{I}{\overline{I}_{ext}}\times 100 \qquad \text{(S2)}$$

Where, I = Gray value of a pixel on a 300 µm vertical line at the end of the serpentine loop,

$\overline{I}_{ext}$= Mean gray value of externally mixed dye in the channel

For green dye solutions, the images were converted to HSB stack and a saturation channel was selected. Next, gray values were plotted along a 300 µm vertical line at the end of the serpentine loop. For each independent experiment, mean gray values were obtained and plotted using ImageJ.

1.4 Primary oral tumor stem-like cell isolation & characterization

Isolation of primary oral tumor cells:

The current study involving human samples was approved by the Institutional Ethics Committee (IEC) of IIT Hyderabad (IEC protocol number: IITH/IEC/2022/04/03). Oral tumor samples from untreated three patients were transported in appropriate medium after obtaining patient's consent in a written format. Isolation of the primary oral tumor cells was performed according to the previously reported procedure, as shown in **Figure S2** [2]. We used mechanical mincing and enzymatic digestion-based method, as optimized before by our group [2]. The cell pellet was suspended in oral tumor growth medium composed of RPMI 1640 + 10% FBS + 1% P/S + epithelial growth factor (EGF) (20 ng/ml) (ProSpec-Tany TechnoGene, Israel) + basic fibroblast growth factor (bFGF) (20 ng/ml) (ProSpec-Tany TechnoGene, Israel)+ B27 (2%) (Invitrogen) [2,3].-The cells were cultured at standard incubation conditions.

Orosphere culture for cancer stem-like cells enrichment:

The isolated cells were trypsinized, and five thousand oral cancer cells were seeded on 1.5% agarose-coated six-well plates. The cells were cultured in low glucose DMEM/F12 supplemented with 2% FBS, 1% P/S, EGF (20 ng/ml), bFGF (20 ng/ml), and B 27 (2%) at standard incubation conditions [2–4]. The images were captured by a light microscope, and counting was performed. The orospheres were dissociated with 1x accutase (Sigma-Aldrich) and characterized by flow cytometry.

Characterization of enriched stem-like tumor cells using flow cytometry:

Characterization of stem-like tumor cells was performed according to the previously reported protocol [2]. Briefly, cells were washed with ice-cold 1x PBS (free from Ca^{+2} and Mg^{+2}), and filtered through a 40 µm cell strainer. Cells (1×10^6) were suspended in 1x PBS and centrifuged at 300×g for 5 min at 4 °C. Pellet was mixed in 100 µl FACS buffer (1x PBS supplemented with 2% FBS) with respective antibodies: CD44-FITC (Elabscience, TX, USA), CD24-PE (Elabscience TX, USA), MSC phenotyping kit (Miltenyi Biotec (#130-125-285).

Staining was performed at 4 °C for 30 min in dark. Following antibody incubation, cells were washed with 1 ml FACS buffer and centrifuged at 300×g for 5 min at 4 °C. Next, the pellet was suspended in 500 µl FACS buffer, and stained cells were evaluated with a flow cytometer (FACSCelesta, BD Biosciences, USA). The data were analyzed using FlowJo software (Beckman Coulter, Inc., Brea, CA, USA).

Immunofluorescence

Indirect immunofluorescence was performed according to the previously reported protocol [2]. Primary antibodies include: epithelial cell adhesion molecule (EpCAM) (1:50), α-smooth muscle actin (αSMA) (1:100), aldehyde dehydrogenase (ALDH1A1) (1:50) (rabbit-anti-human, Elabscience, TX, USA). Followed by overnight 4 °C incubation with primary antibodies, the species-specific fluorochrome-conjugated goat-anti-rabbit-IgG Alexa Fluor 488 secondary antibody (1:200, Elabscience, TX, USA) was added for 45 min. The cells were counterstained with nuclear stain DAPI (1 µg/ml, Sigma-Aldrich). After incubation, the cells were washed gently with 1x PBS and visualized under EVOS M7000 fluorescent microscope.

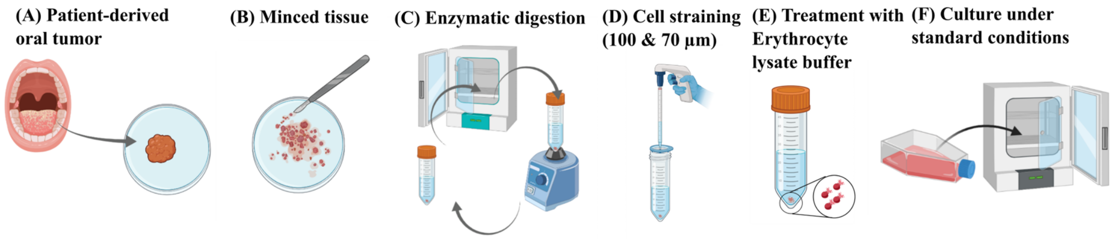

Figure S2. Isolation protocol of oral tumor cells: (A) Patient-derived oral tumor biopsy sample is obtained. (B) Oral tumor sample is minced in 2-3 mm pieces. (C) Enzymatic digestion is carried out using collagenase and hyaluronidase. (D) Next, the cell suspension is filtered through 100 and 70 µm cell strainers. (E) Treatment with Ammonium-Chloride-Potassium (ACK) lysing buffer (5 min) at room temperature to remove red blood cells (RBCs) from the cell mixture. (F) Cells are cultured under standard incubation conditions. Finally, isolated oral

tumor cells are cultured in orosphere format to enrich oral stem-like cell population. This illustration has been prepared with BioRender.com

2. Supplementary results

Figure S3 shows the overall sequence in which experiments for the tumor-on-chip were performed and presented in the manuscript.

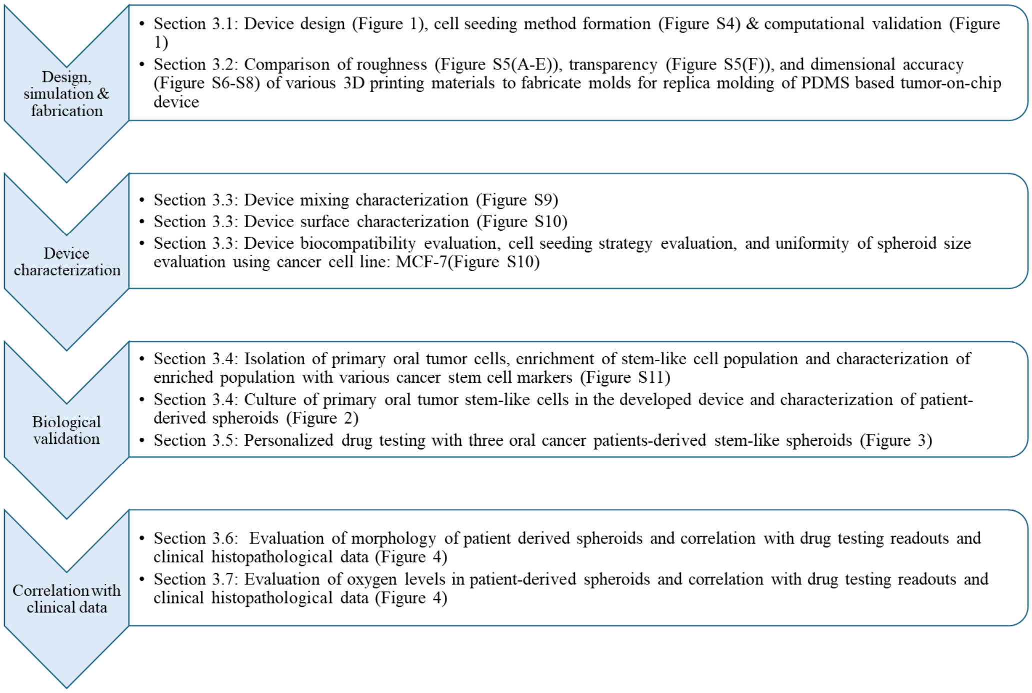

Figure S3. Flow-chart showing the sequence of experimental results presented in this manuscript

2.1 Cell seeding method for uniform-sized spheroids formation

The optimized cell seeding is a six-step process as described below:

1. Tumor cells (6000/µl, 20 µl suspension) are introduced through 1 ml tips from each opening of serpentine loop at 12 µl/min using a syringe pump. The syringe pump withdraws fluid from three removable ports (RP) by applying a negative pressure for 2 min (**Figure S4 (A(i))**).

2. Subsequently, the syringe pump is stopped to allow cells to settle at the bottom of each microwell array and milliwells (**Figure S4 (A(ii))**). Based on the input cell concentration of 6000 cells/µl/microwell array, the estimated number of cells captured in each microwell with a volume of 0.05 µl will be approximately 300 cells. In this 2nd step, the excess cells in the 1 ml tips settle down in 7 milliwells located under each inlet port (IP) and outlet port (OP), thereby preventing them from entering the channel network.

3. Next, the silicon tubes are trimmed by keeping tube luers inserted in RP (**Figure S4 (A(iii))**). At the end of 3rd step, the *untrapped* cells will be lying throughout the channel network of the device. If these *untrapped* cells are not removed, they may be carried into the microwells by fluid movement, thereby disrupting the uniformity of the cell seeding process. Additionally, fluid movement following cell seeding may cause cells that have been captured in the microwells to be dislodged.

4. Hence, the device is kept in static incubation for 24 h for spheroid formation without causing any fluid movement after cell seeding (**Figure S4 (A(iv))**).

5. After 24 h, each microwell will contain a spheroid. Moreover, some of the cells residing in the channel network will also make small aggregates as the whole device is coated with pluronic F127. We capture these *untrapped* small aggregates and cells by inducing fluid flow towards RP, thereby preventing them from entering the microwells. This is achieved by detaching silicon tube luers from RP, which induces a gravity-based fluid flow. This flow captures loose cells and aggregates from the channel network, directing them into an additional set of three milliwells located beneath each silicone tube luer (or RP) (**Figure S4 (A(v))**). As

the excess cells and aggregates are washed out, the device is left with single spheroid in each microwell with uniform size. Without the help of the 5th step, these *untrapped* aggregates and cells would have entered the microwells during media change or drug perfusion, thereby disturbing the uniform size of spheroids.

6. On day 3, spheroids are exposed to drugs inside an incubator with a flow rate of 2 µl/min (**Figure S4 (A(vi))**). The device remains inside the 3D printed enclosure, which has a slot to allow silicon tubes to pass through it.

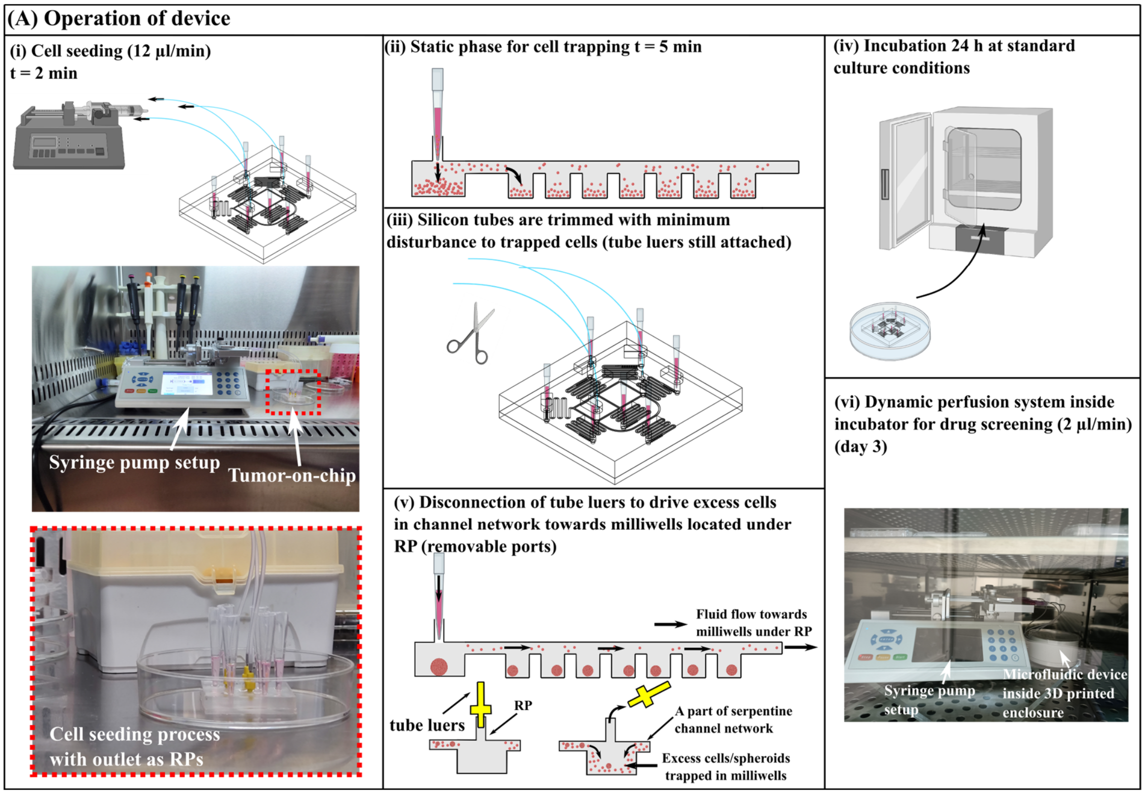

Figure S4. (A) Operation of device: (i) Cell seeding is performed with a syringe pump at 12 µl/min for 2 min, (ii) After the cell seeding, pump is stopped and cells settle down in microwells within 5 min of static phase. In 2nd step, the excess cells in the 1 ml tips settle down in 7 milliwells located under each IP and OP, thus preventing them from entering the channel network, (iii) Next, silicon tubes are trimmed to disconnect the syringe pump from the device, while keeping silicon tube luers attached to RP, (iv) Device is kept in static incubation for 24

h to form spheroids, (v) Tube luers are disconnected from chip to capture excess cells and aggregates from channel network in milliwells located under RPs (removable ports), (vi) Finally, single spheroids in each microwell are formed and cultured over a period of 3 days. Spheroids are exposed to drugs inside an incubator with a 2 µl/min flow rate on day 3.

2.2 Comparison of resins

PDMS devices fabricated from Accura 25 and Accura ClearVue possessed better surface finish compared to other resins (**Figure S5(A-E)**). This was also reflected in the optical transparency of PDMS devices (**Figure S5(F)**). We found all resins except Accura 25 and Accura ClearVue had average surface roughness of more than 1 µm and mean optical transparency of less than 90%. However, all PDMS devices exhibited a mean transparency of more than 85%, indicating that molds made from all five photocurable resins are suitable for fabricating transparent replica molded PDMS microfluidic devices. Hence, we further considered dimensional accuracy in determining the best photocurable resin.

Next, we measured the PDMS channel dimensions fabricated from each resin mold, as shown in **Figure S6(A)**. Interestingly, measured width values for each resin varied linearly with the designed width (**Figure S6(B-F)**). Moreover, 3D-printed channels deviated from the designed channel width due to tapered channel walls. In addition to the tapered walls, channels printed in MJP had rounded bottom, thus deviating significantly from the designed rectangular cross-section (Figure S6(F)). Hence, MJP printed devices were not considered further in the comparative study.

We specifically compared the printed width of the 400 µm channels, as the device in the current study has a channel network with a rectangular cross-section of 400 × 200 µm. We found that deviation in measured width was significantly lower in Formlabs standard clear and Accura ClearVue than other resins (**Figure S7(A)**). Lower channel dimensional variation is

necessary, as the drug diffusion depends on the channel width. We also measured deviation in printed microwells in all five resins. We found that measured diameter values of microwells also varied linearly with the designed diameter in all resins (**Figure S8(B-F)**). Besides, well sizes less than 300 µm could only be printed with Formlabs standard clear and Accura ClearVue. Based on the overall comparison, we considered Formlabs standard clear and Accura ClearVue for further evaluation by fabricating soft-lithography molds for spheroids-on-a-chip.

We fabricated soft-lithography molds of the designed device from Formlabs standard clear and Accura ClearVue reins. Since the diameter of the spheroids depends on the diameter of the printed microwells, we measured all forty-nine microwells, each designed with a diameter and height of 400 µm. We found that standard clear resin produced a significant variation in well diameter with mean diameter of 308 µm and standard deviation of 2.467 %. (**Figure S7(B)**).

Variation in the height of microwells in Accura ClearVue was significantly higher, with a standard deviation of 6.354% and a mean height of 320 µm. We experienced that spheroid in a microwell with a height of less than 320 µm would escape out while performing media exchange or silicon tube insertion/removal (data not shown). Hence, the standard clear resin was finalized for printing soft-lithography molds for spheroids-on-a-chip (**Figure S7(C)**). As the standard deviation in diameter values was low (2.467 %), we hypothesized that the spheroid sizes would be uniform across the seven PDMS microwell arrays fabricated from standard clear resin mold. **Figure S7(D)** shows the assembled PDMS spheroids-on-a-chip used for combinatorial drug screening.

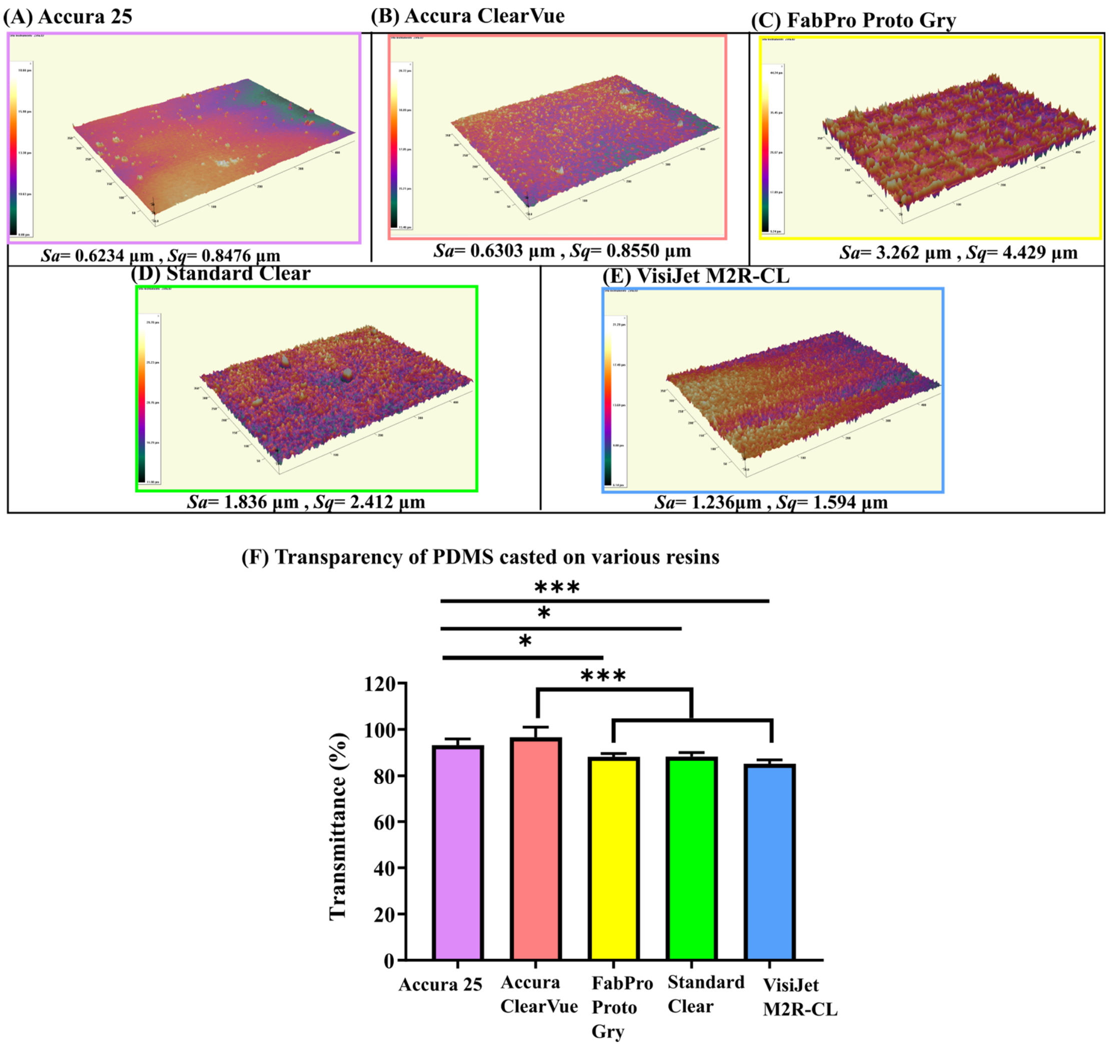

Figure S5. (A-E) Surface roughness parameters of casted PDMS on various resins. (F) Optical transmittance (%) of casted PDMS on various resins. (Mean ± SD, A one-way ANOVA, Tukey's post hoc test, n=6, p<0.05(*), p<0.001(***)).

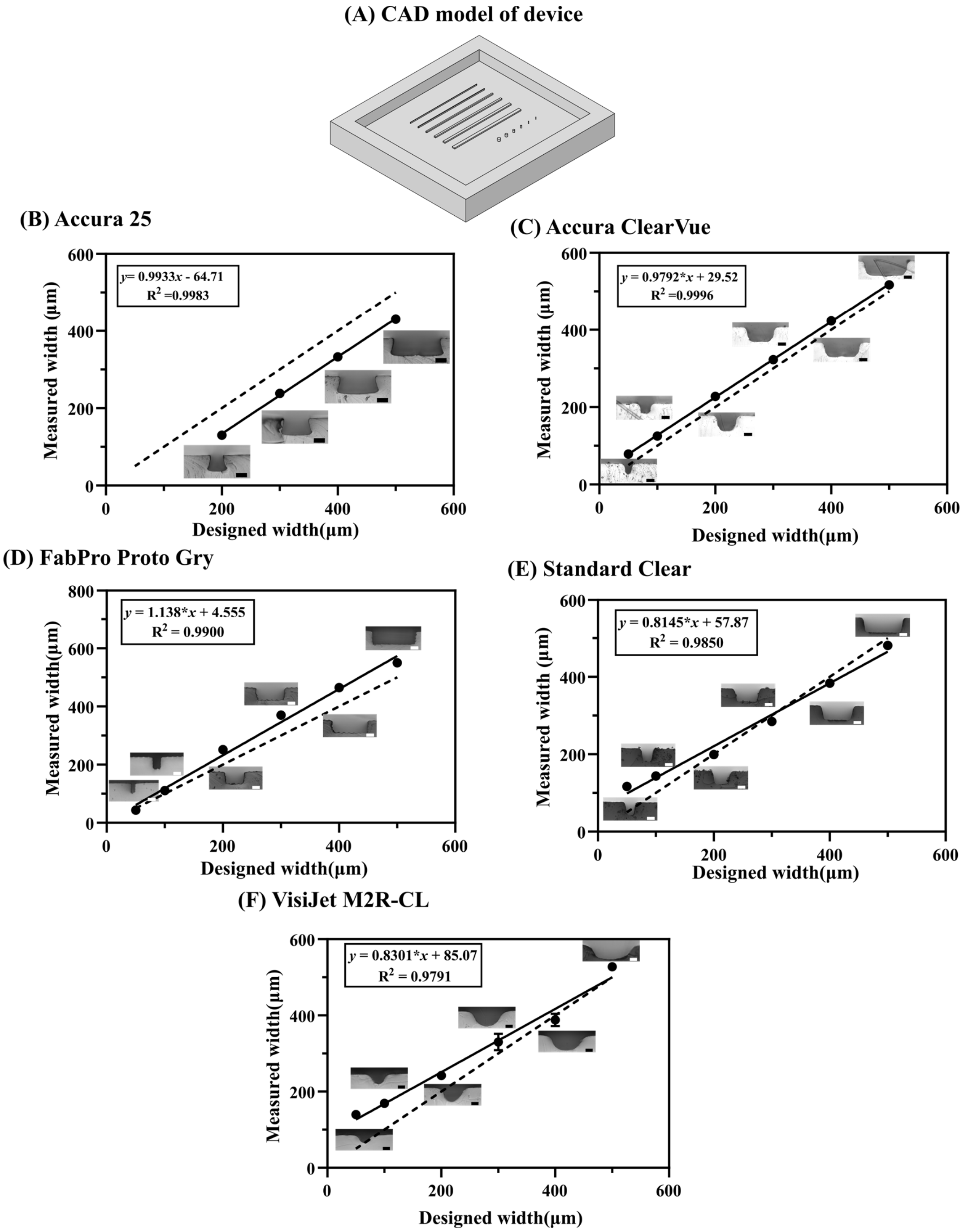

Figure S6. (A) CAD model showing the design of the mold used for the resolution study. (B-F) Variation in width of PDMS channels fabricated by replica molding over various 3D printed resin molds. (the dotted line shows an ideal case where measured width precisely matches designed width). (Mean ± SD, *n*=2) (scale bar = 100 µm)

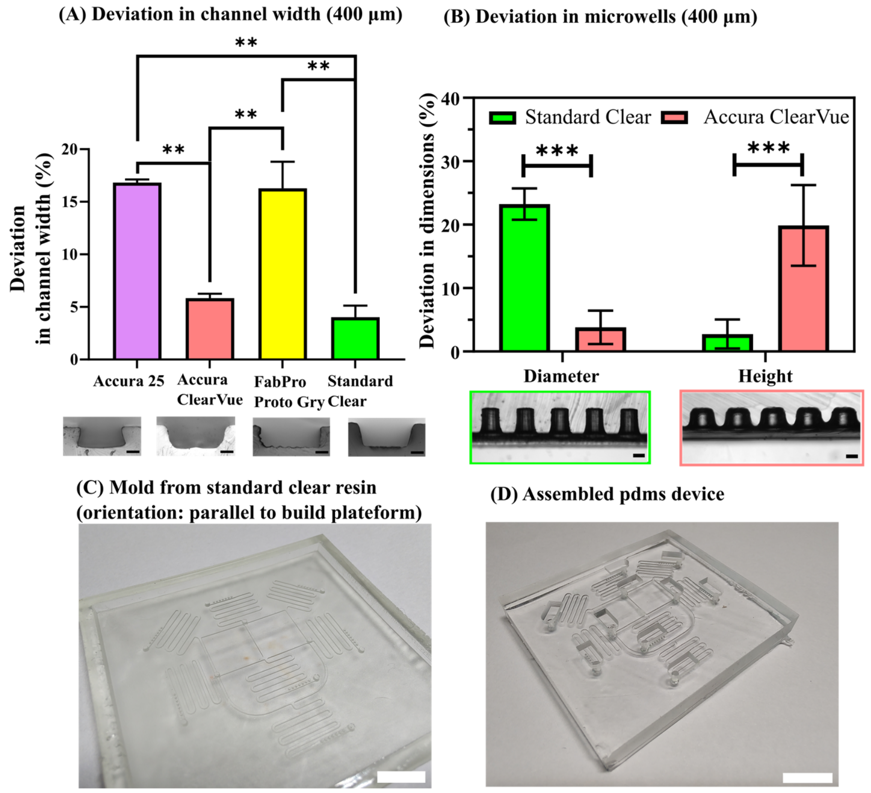

Figure S7. (A) Deviation in printed channels of 400 µm width (Mean ± SD, A one-way ANOVA, Tukey's post hoc test, n=2, p<0.01(**)) (scale bar = 100 µm). (B) Deviation in height and diameter of all forty-nine microwells printed in standard clear and Accura ClearVue resins. (Mean ± SD, student's t-test, p<0.001(***)) (scale bar = 200 µm). (C) Standard clear resin mold printed parallel to the build platform and used for fabricating PDMS-based spheroids-on-a-chip (scale bar = 10 mm). (D) Assembled PDMS device using plasma bonding (scale bar = 10 mm).

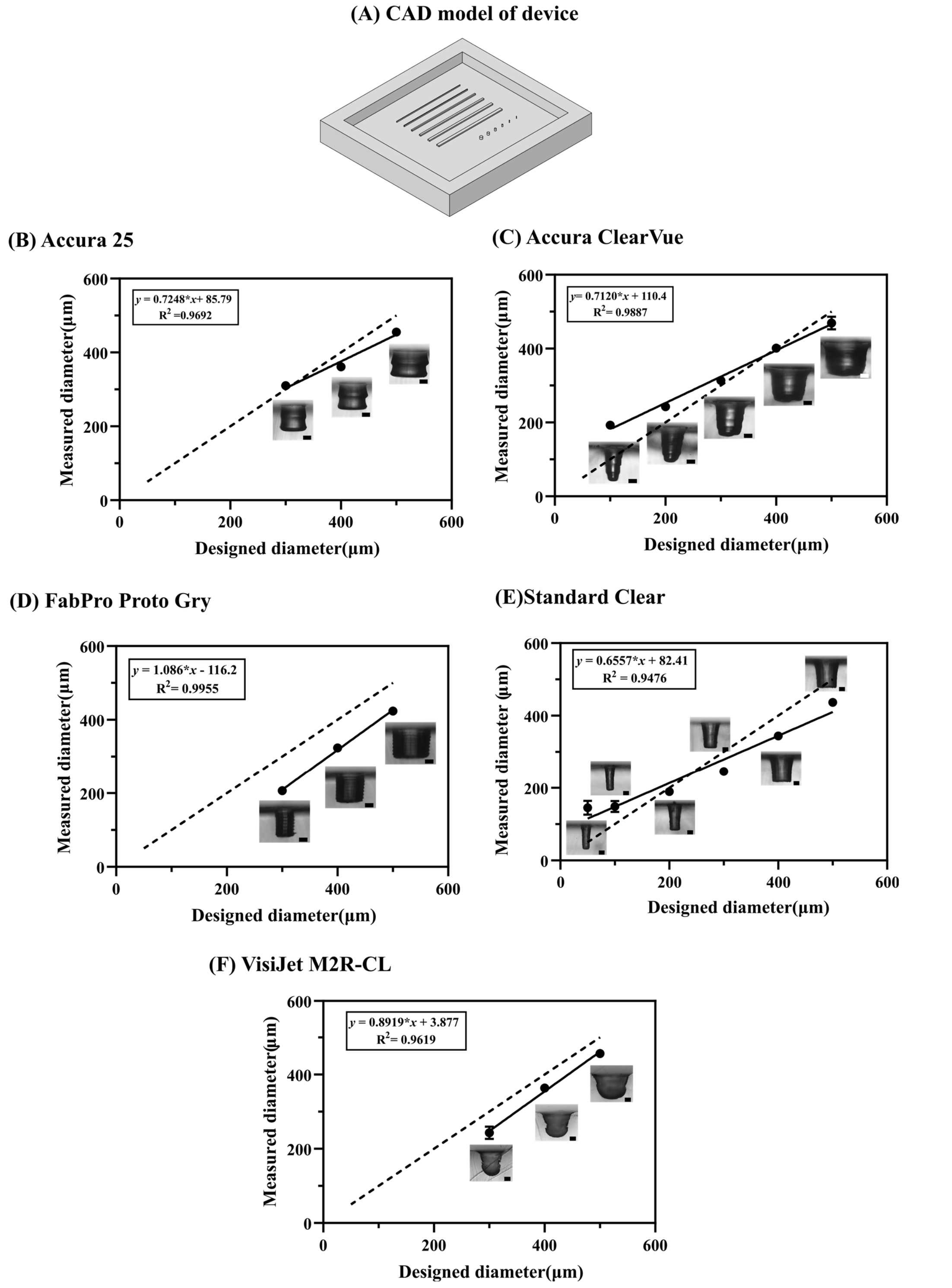

Figure S8. (A) CAD model showing the design of mold used for resolution study. (B-F) Variation in diameter of PDMS microwells fabricated by replica molding over various resin molds. (the dotted line shows an ideal case where measured diameter exactly matches designed diameter) (Mean ± SD, n=2) (scale bar = 100 µm)

2.3 Device characterization and validation with tumor cell line

Figure S9(A) depicts the device in its dynamic phase, displaying an interface of color dyes in the bottom-most serpentine loop and interface between color dyes and water in the middle serpentine loop. **Figure S9(B)** shows the mixing (%) plotted along a 300 μm line at the end of the serpentine loop containing blue solution. We achieved experimental mixing (%) of 96.54 ± 0.063%, which closely matched the average mixing (%) of 98.3% obtained from simulation results.

Given that the water stream merges with the color dyes in the middle-most serpentine loop, the mean gray value of the green dye solution in this loop is expected to be lower than that of the green solution emerging from the bottom-most loop. We plotted gray values at the end of middle-most and bottom-most serpentine loops along a 300 μm vertical line (**Figure S9(C)**). We found that the mean gray values for both solutions were significantly different ($p < 0.01$). We obtained 91.79 ± 1.374 mean gray value for middle-most loop, which was lower than mean gray value of 101.09 ± 1.058 calculated for bottom-most loop.

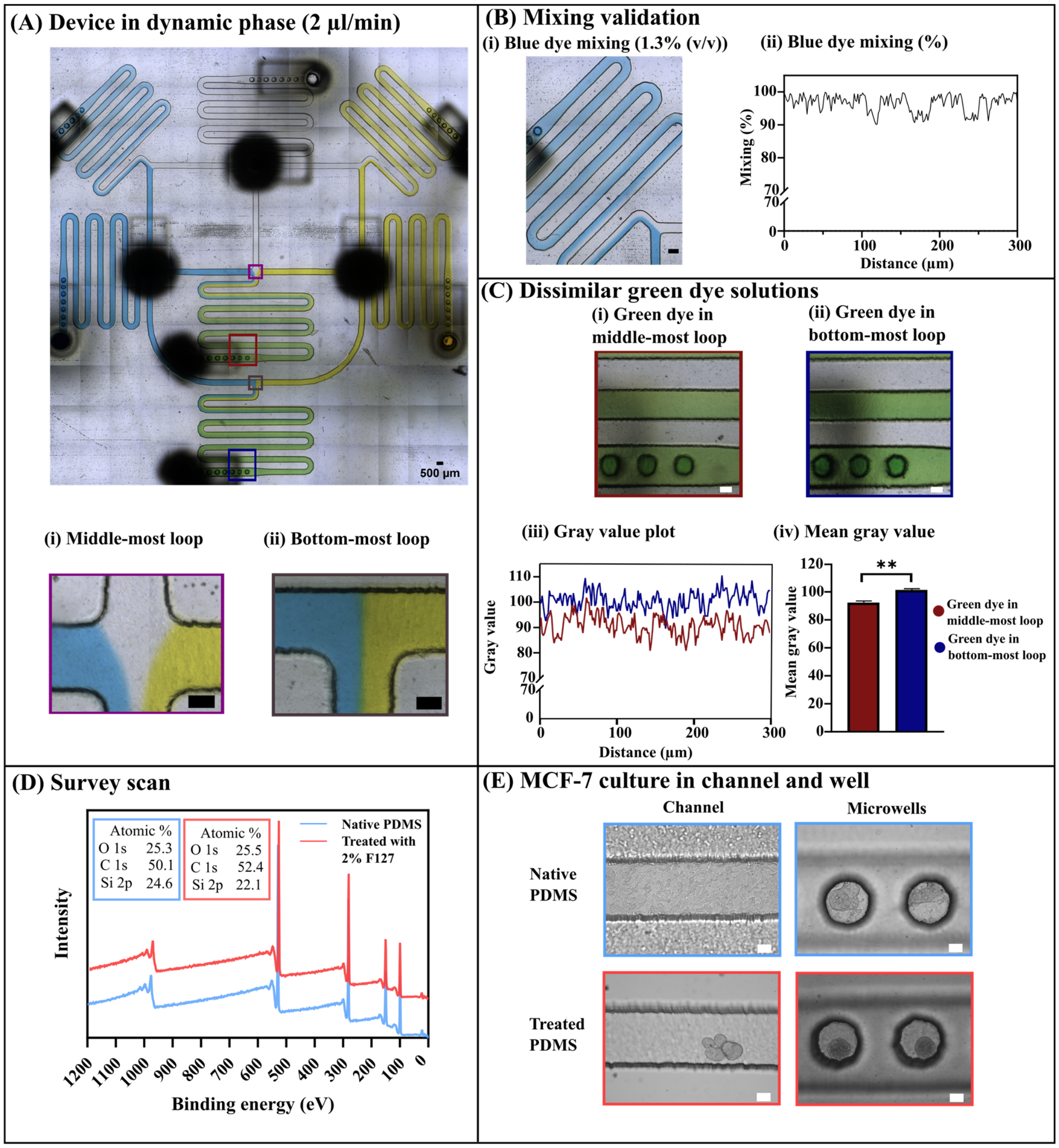

Figure S9. Device mixing (%) characterization: (A) Color dyes and water are withdrawn at 2 µl/min, shown in a brightfield image during dynamic phase (scale bar = 200 µm). (B) Characterization of mixing (%): (i) Brightfield image showing blue dye solution (1.3% v/v) and water interface in a serpentine loop in a dynamic phase (scale bar = 500 µm), (ii) Variation in mixing (%) along a 300 µm line at the end of serpentine loop. (C) Dissimilar green dye solutions: (i) Brightfield image showing green dye solution at the end of the middle-most serpentine loop (scale bar = 200 µm), (ii) Brightfield image showing green dye solution at the end of the bottom-most serpentine loop (scale bar = 200 µm), (iii) Gray value variation along

a 300 μm vertical line at the end of both serpentine loops containing green dye solutions, (iv) Mean gray value comparison for both green dye solutions. The mixing experiment was repeated four times (*n*=4), and results were presented as Mean ± SEM. The mean gray values for two green dye solutions from mixing loops were compared using Student's t-test (D) XPS Survey scan of native and coated PDMS. (E) MCF-7 cell culture on coated and native PDMS devices (scale bar = 100 μm).

Furthermore, we performed surface characterization of the PDMS device after applying 2% (w/v) coating of pluronic F127 for 24 h, intended to induce cell aggregation using XPS and contact angle goniometer. C1s XPS analysis of native and treated PDMS surface demonstrated one major peak at 284.8 eV due to $(CH_3)_2SiO$ groups present in PDMS [5]. In addition, coated PDMS showed a minor peak at 286.6 eV, suggesting the presence of CH_2CH_2O group found in pluronic F127 [6,7] (**Figure S10(B)**). In line with C1s spectra, the survey scan showed an increase in the amount of atomic carbon and oxygen due to presence of pluronic groups (**Figure S9(D)**). Moreover, the presence of pluronic groups rendered PDMS surface hydrophilic (~56°), whereas native PDMS remained hydrophobic (~95°) (**Figure S10(C)**). The pluronic-coated hydrophilic surface is beneficial in microfluidics, as it minimizes air bubble formation during fluid perfusion.

A breast cancer cell line (MCF-7) was cultured in the PDMS device to assess biocompatibility, uniform-sized spheroid formation with the developed cell seeding method (Figure S4), standard clear resin mold-based fabrication strategy (main manuscript section 3.2), and pluronic F127 based coating strategy. The breast cancer cell line was used only to evaluate the above mentioned details and not to perform any drug screening. As shown in **Figure S10(D)**, uniform-sized spheroids could be formed in all microwells. We found the spheroid size remained under 200 μm during 6 days of culture with a maximum standard deviation of

11.97 μm on day 6 (**Figure S10(E)**). Moreover, mean spheroid size was found increasing during 6 days of culture. The mean circularity index of 0.95 shows the excellent capability of 3D printing-based microwell arrays to form circular spheroids (**Figure S10(F)**). We had hypothesized that a low standard deviation in measured microwell sizes would enable formation of uniform-sized spheroids in PDMS device fabricated from standard clear resin mold (section 3.2). Our MCF 7 spheroid size results corroborate this hypothesis. Finally, MCF-7 spheroids maintained greater than 90% viability, proving the device's excellent biocompatibility (**Figure S10(G,H)**).

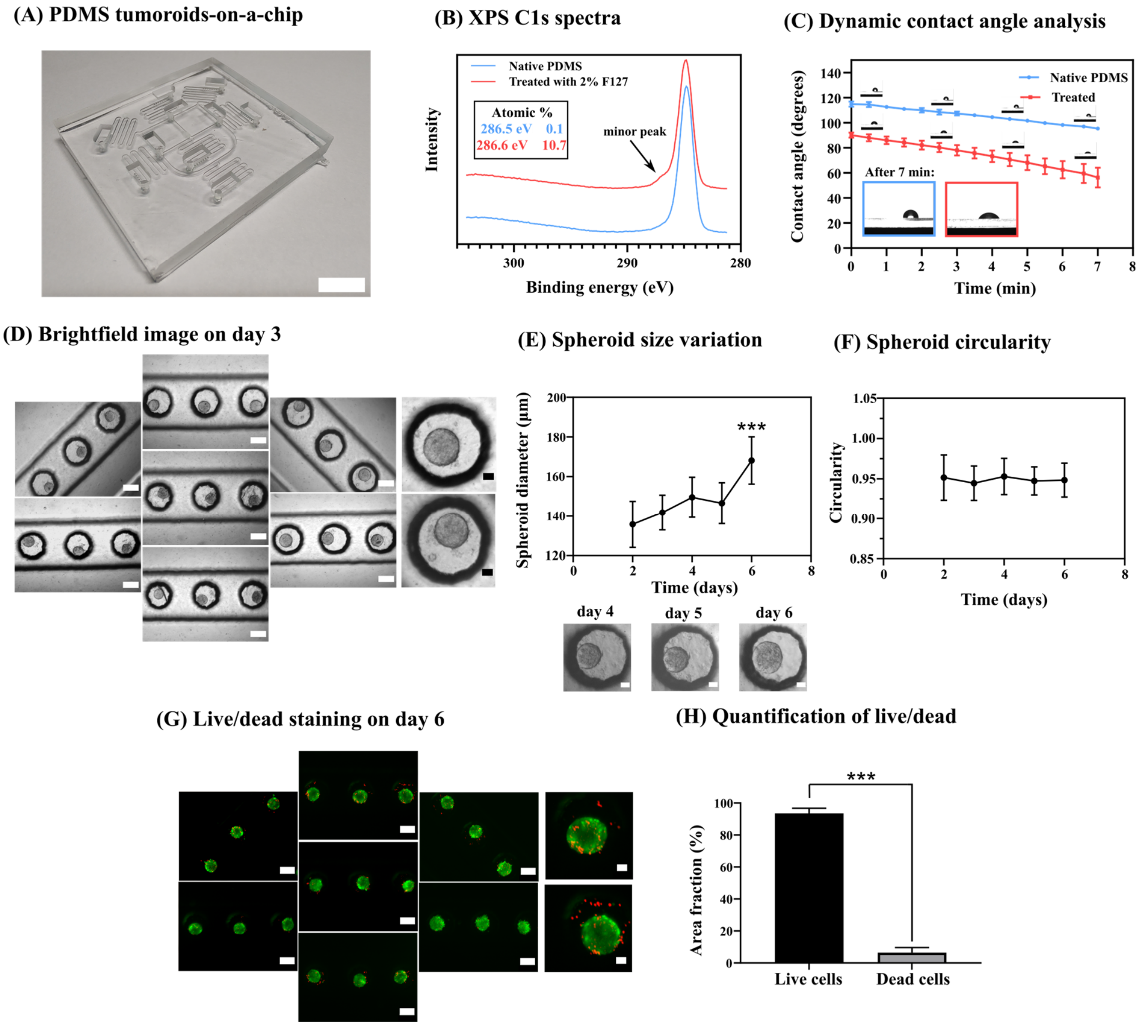

Figure S10. (A) Assembled PDMS spheroids-on-a-chip used for cell culture and drug screening (scale bar = 10 mm). (B) XPS C1s spectra of native and coated PDMS devices showing pluronic groups in coated group. (C) Further validation of pluronic F127 presence by dynamic contact angle analysis. (Mean ± SD, *n*=3) (D) MCF-7 cells are seeded with a 12 µl/min flow rate, and brightfield images of spheroids are captured on day 3 (left panel scale bar = 200 µm and right magnified panel scale bar = 50 µm). (E) Spheroid size variation measured with time using ImageJ. (Mean ± SD, Student's t-test, $p<0.001$ (***) with day 2, *n*=49 spheroids) (scale bar = 50 µm) (F) Spheroid circularity measured with time using ImageJ (Mean ± SD, *n*=49 spheroids). (G) Live/dead staining showing live cells in green and dead cells in red on day 6 (scale bar in left panel = 200 µm and right magnified panel = 50 µm). (H) Quantification

of live/dead staining on day 6. (Mean ± SD, Student's t-test, $p < 0.001$ (***), n=49 spheroids). The spheroids cultured in left-most and right-most microwell arrays have been shown in the horizontal orientation.

3. Supplementary figures & tables

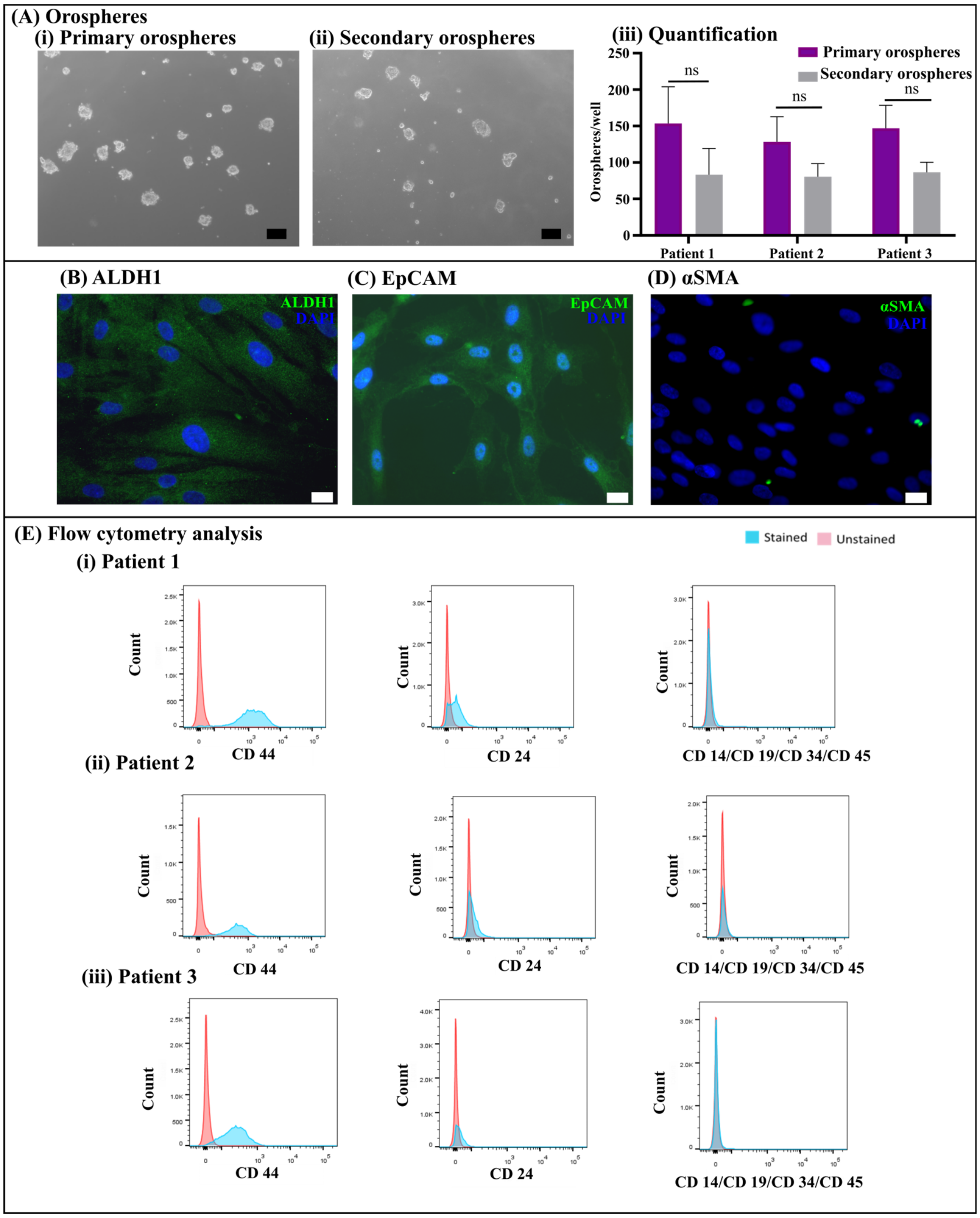

Figure S11. (A) Orosphere assay to prove the self-renewal capacity of isolated tumor cells: (i) Brightfield image of primary orospheres (scale bar = 100 μm), (ii) Brightfield image of secondary orospheres, (iii) Quantification of primary and secondary orospheres per well (mean ± SD, *n*=3, student's t-test) (B) ALDH1 staining showing positive cells in green color (scale bar = 20 μm). (C) EpCAM expression showing positive cells in green color (scale bar = 20 μm). (D) αSMA expression (scale bar = 20 μm). (E) Flow cytometry analysis of isolated oral tumor stem-like cells showing cancer stem cell markers CD 44^{High} and CD 24^{Low} for: (i) Patient 1, (ii) Patient 2, (iii) Patient 3.

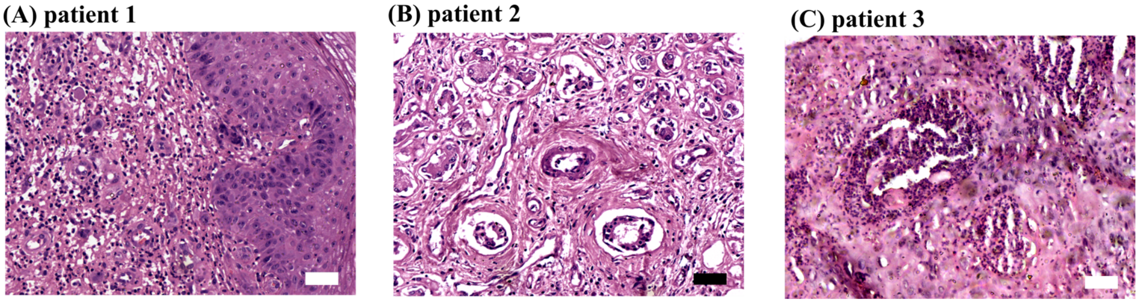

Figure S12. H&E staining of patient biopsy samples: (A) Patient 1. (B) Patient 2. (C) Patient 3 (scale bar = 50 μm).

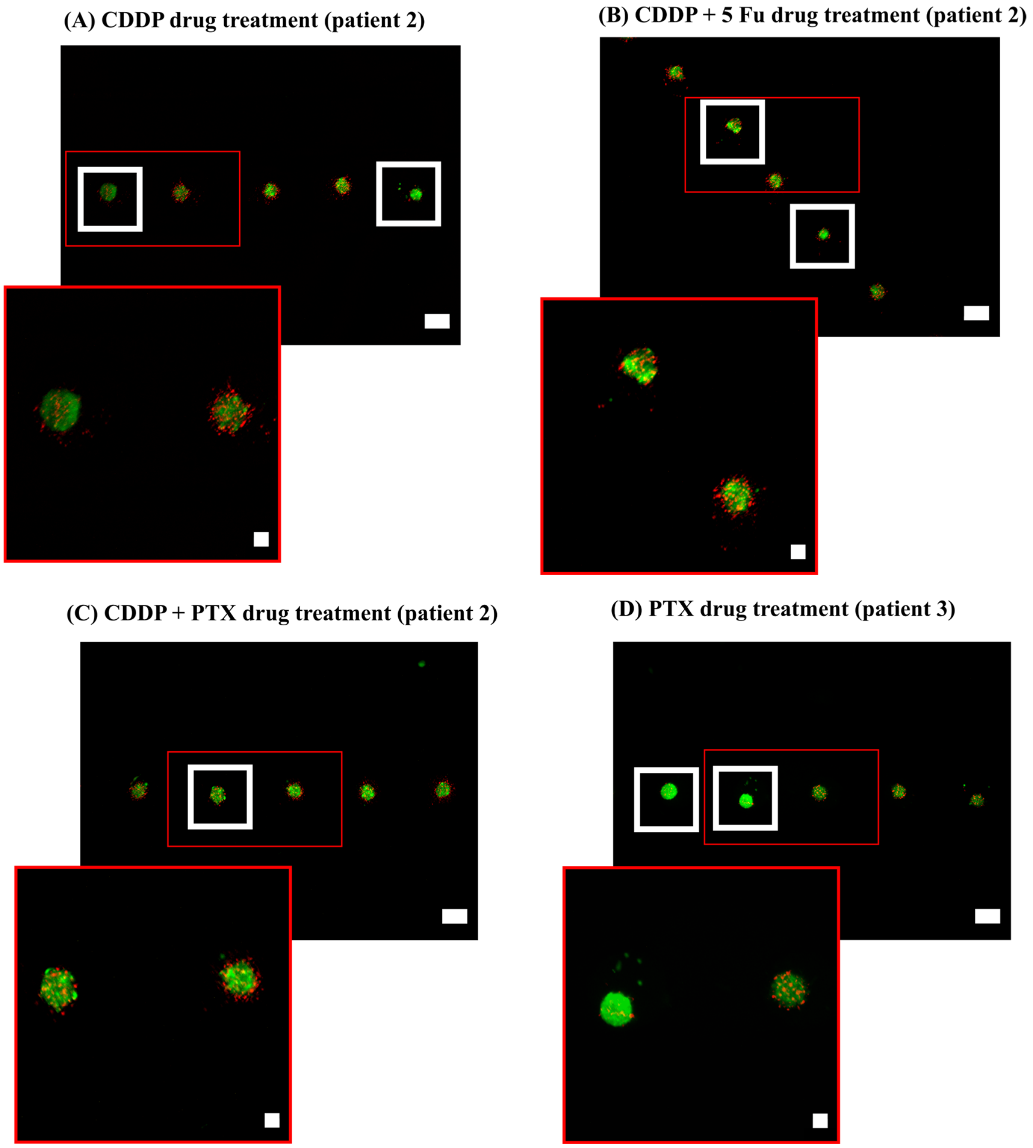

Figure S13. Spheroid arrays showing several spheroids with reduced chemosensitivity towards drug treatments (shown by white boxes): (A) Cisplatin drug treatment in patient 2. (B) Cisplatin + 5 Fu drug treatment in patient 2. (C) Cisplatin + paclitaxel drug treatment in patient 2. (D) Paclitaxel drug treatment in patient 3. (Scale bar = 100 μm and scale bar in magnified panels = 50 μm).

Table S2. Comparison of the present technology with existing 3D spheroids-on-chips platforms

Cancer type (cell source)	Study objective (fabrication method)	No. of patient samples reported	Clinical correlation of drug responses	Oxygen levels measurement	Number of spheroids & (drugs or drug combinations tested per experiment, per chip)	End-point analysis method	Study outcome	Reference (year of publication)
Breast cancer (cell lines)	Nanoparticles uptake evaluation in 3D spheroids-on-chips (photolithography)	NA	NA	NR	256 (1)	Imaging	Dynamic systems provide better uptake evaluation	[8] (2019)
Colorectal cancer (PDX cell line)	To compare performance of spheroid-on-chip with PDX animal models (photolithography)	3	NR (Comparison with animal PDX model reported, spheroid morphological changes not reported, no correlation with histopathologica l data)	NR	32 (1)	Imaging	Results correlated fairly with *in vivo* PDX model for 2 patients	[9] (2022)
Variety of cancer types (no oral cancer samples)	personalized drug screening (3D printing)	10	Very few direct correlations reported, spheroid morphological	NR	Multiple spheroids were tested (*n*=48) with 1 drug or drug	Imaging	Study reported very few direct correlations	[10] (2023)

(primary cells isolated from fresh tumor)			changes not reported, no correlation with histopathological data		combination per experiment and per chip		to patient outcomes	
Prostate cancer (primary cells isolated from fresh tumor)	Personalized drug screening (photolithography)	2	NR	NR	240 (1)	Imaging	Proof-of-concept was shown successfully	[11] (2018)
Ovarian cancer (PDX cell line)	Personalized drug screening (photolithography)	6	NR	NR	11 (1)	Imaging	Proof-of-concept was shown successfully	[12] (2020)
Pancreatic cancer (PDX cell line)	Personalized drug screening (photolithography)	3	NR	NR	1032 (8)	Imaging	Study successfully reported one of the most complex devices to screen 172 different conditions.	[13] (2018)
Breast cancer (primary cells isolated from fresh tumor)	Personalized drug screening (photolithography)	2	NR (Comparison for patient 1 was provided with *in vivo* PDX model, spheroid morphological changes not	Reported (HIF1α)	800 (1)	Imaging	Patient 1 data showed correlation with *in vivo* PDX drug testing data	[14] (2022)

			reported, no correlation with histopathologica l data)					
Lung cancer (cell lines)	Molecular targeted therapies testing (3D printing)	NA	NA	NR	1000 (1)	Imaging	3D spheroids showed higher drug resistance compared to 2D monolayer	[15] (2022)
Lung cancer (primary cells isolated from fresh tumor)	Personalized drug screening (3D printing)	2	NR	NR	8 (1)	Imaging	Higher resistance in primary co-culture spheroids than mono-culture spheroids	[16] (2015)
Variety of cancer cell lines	To compare static vs dynamic culture of spheroids (photolithography)	NA	NA	NR	Multiple microwells with 1 drug testing per chip, per experiment	Imaging	Better spheroid growth, morphologica l features, and higher drug resistance in dynamic culture than static culture.	[17] (2024)
Oral cancer	Personalized drug screening	3	Compared the *in vitro* drug	Reported	7 (3)	Imaging	Oral tumoroids	Present work (2024)

(primary cells isolated from fresh tumor)	(3D printing)		testing data with patient histopathologica l data				exhibit dissimilar morphology, size, and oral tumor-relevant oxygen levels (< 5% O2). These features correlate with the drug responses and clinical diagnosis from each patient's histopatholog ical report.	
NR: Not reported, NA: Not applicable								

References

1. Mehta V, Sudhakaran SV, Rath SN. Facile Route for 3D Printing of Transparent PETg-Based Hybrid Biomicrofluidic Devices Promoting Cell Adhesion. ACS Biomater Sci Eng. 2021;7:3947–63.

2. S. S V, Giri PS, Nellore V, Rath SN. Osteomatrix as a personalized 3D tissue-specific invasion test-bed for oral carcinoma. Biomater Sci [Internet]. 2023;11:4265–80. Available from: https://pubs.rsc.org/en/content/articlehtml/2023/bm/d2bm01870e

3. Kim HS, Pearson AT, Nör JE. Isolation and characterization of cancer stem cells from primary head and neck squamous cell carcinoma tumors. Methods Mol Biol [Internet]. 2016 [cited 2022 May 5];1395:241. Available from: /pmc/articles/PMC5344035/

4. Krishnamurthy S, Nör JE. Orosphere assay: A method for propagation of head and neck cancer stem cells. Head Neck. 2013;35:1015–21.

5. Ren W, Ai Z, Jia F, Zhang L, Fan X, Zou Z. Low temperature preparation and visible light photocatalytic activity of mesoporous carbon-doped crystalline TiO2. Appl Catal B. 2007;69:138–44.

6. Pinto S, Alves P, Matos CM, Santos AC, Rodrigues LR, Teixeira JA, et al. Poly(dimethyl siloxane) surface modification by low pressure plasma to improve its characteristics towards biomedical applications. Colloids Surf B Biointerfaces. 2010;81:20–6.

7. Wu Z, Hjort K. Surface modification of PDMS by gradient-induced migration of embedded Pluronic. Lab Chip [Internet]. 2009 [cited 2022 Jul 18];9:1500–3. Available from: https://pubs.rsc.org/en/content/articlehtml/2009/lc/b901651a

8. Zhuang J, Zhang J, Wu M, Zhang Y, Zhuang J, Zhang J, et al. A Dynamic 3D Tumor Spheroid Chip Enables More Accurate Nanomedicine Uptake Evaluation. Advanced Science [Internet]. 2019 [cited 2023 Sep 21];6:1901462. Available from: https://onlinelibrary.wiley.com/doi/full/10.1002/advs.201901462

9. Ong LJY, Chia S, Wong SQR, Zhang X, Chua H, Loo JM, et al. A comparative study of tumour-on-chip models with patient-derived xenografts for predicting chemotherapy efficacy in colorectal cancer patients. Front Bioeng Biotechnol. 2022;10:952726.

10. Steinberg E, Friedman R, Goldstein Y, Friedman N, Beharier O, Demma JA, et al. A fully 3D-printed versatile tumor-on-a-chip allows multi-drug screening and correlation with clinical outcomes for personalized medicine. Communications Biology 2023 6:1 [Internet]. 2023 [cited 2024 May 14];6:1–14. Available from: https://www.nature.com/articles/s42003-023-05531-5

11. Mulholland T, McAllister M, Patek S, Flint D, Underwood M, Sim A, et al. Drug screening of biopsy-derived spheroids using a self-generated microfluidic concentration gradient. Sci Rep [Internet]. 2018;8:14672. Available from: /pmc/articles/PMC6168499/

12. Dadgar N, Gonzalez-Suarez AM, Fattahi P, Hou X, Weroha JS, Gaspar-Maia A, et al. A microfluidic platform for cultivating ovarian cancer spheroids and testing their responses to chemotherapies. Microsyst Nanoeng [Internet]. 2020;6:1–12. Available from: www.nature.com/micronano

13. Zhang Z, Chen YC, Urs S, Chen L, Simeone DM, Yoon E. Scalable Multiplexed Drug-Combination Screening Platforms Using 3D Microtumor Model for Precision Medicine. Small [Internet]. 2018;14:1703617. Available from: http://doi.wiley.com/10.1002/smll.201703617

14. Prince E, Kheiri S, Wang Y, Xu F, Cruickshank J, Topolskaia V, et al. Microfluidic Arrays of Breast Tumor Spheroids for Drug Screening and Personalized Cancer Therapies. Adv Healthc Mater. 2022;11.

15. Luan Q, Becker JH, Macaraniag C, Massad MG, Zhou J, Shimamura T, et al. Non-small cell lung carcinoma spheroid models in agarose microwells for drug response studies. Lab Chip [Internet]. 2022 [cited 2024 May 14];22:2364–75. Available from: https://pubs.rsc.org/en/content/articlehtml/2022/lc/d2lc00244b

16. Ruppen J, Wildhaber FD, Strub C, Hall SRR, Schmid RA, Geiser T, et al. Towards personalized medicine: Chemosensitivity assays of patient lung cancer cell spheroids in a perfused microfluidic platform. Lab Chip. 2015;15:3076–85.

17. Tian D, Mao Z, Wang L, Huang X, Wang W, Luo H, et al. Rocking- and diffusion-based culture of tumor spheroids-on-a-chip. Lab Chip [Internet]. 2024 [cited 2024 May 14];24:2561–74. Available from: https://pubs.rsc.org/en/content/articlehtml/2024/lc/d3lc01116j
